# The impact of improving haemophilia A management within the Spanish National Healthcare System: a social return on investment analysis

**DOI:** 10.1186/s12913-021-07447-4

**Published:** 2022-01-26

**Authors:** Inmaculada Soto, José Mateo, Daniel-Aníbal García-Diego, Beatriz Gil, Elena Ruiz-Beato, Yoana Ivanova, Teresa Martín Lorenzo, Paulina Maravilla-Herrera, Álvaro Hidalgo-Vega, María Merino

**Affiliations:** 1 Hospital Universitario Central de Asturias, Oviedo, Spain; 2 Hospital de la Santa Creu i Sant Pau, Barcelona, Spain; 3 Federación Española de Hemofilia – FEDHEMO, Madrid, Spain; 4 Roche Farma España, Madrid, Spain; 5 Weber, Calle Moreto, 17, 5 Dcha, 28014 Madrid, Spain; 6 Universidad de Castilla-La Mancha, Toledo, Spain

**Keywords:** Disease management, Economic evaluation, Social impact, Public health, Rare disease, Spain

## Abstract

**Background:**

Haemophilia A (HA) has been associated with poor health-related quality of life and a large economic burden, accentuated by severity, arthropathy, and inhibitors. To meet global standards of care, the management of HA should align with the principles of care outlined by the World Federation of Haemophilia. The aims of the present study were to establish a set of proposals to improve HA management within the Spanish National Health System (SNHS) and to estimate the impact its hypothetical implementation would generate from a clinical, healthcare, economic, and social perspective.

**Methods:**

A multidisciplinary group of experts agreed on a set of 15 proposals to improve HA management within the SNHS. Thereafter, a forecast-type Social Return on Investment analysis was carried out to estimate the impact of implementing this set of proposals within the SNHS over a one-year timeframe, in relation to the required investment.

**Results:**

This study estimated that the implementation of the complete set of 15 proposals would require a total investment of 2.34 M€ and have a total impact of 14.60 M€. Accordingly, every euro invested in the complete set of 15 proposals would yield a social return of €6.23 (€3.37 in the worst-case scenario and €9.69 in the best-case scenario) of both tangible and intangible nature in similar proportions (45.71 and 54.29%, respectively).

**Conclusions:**

These results can be used to inform policy and practice such that interventions that may potentially improve current public health challenges associated with the management of HA may be implemented.

**Supplementary Information:**

The online version contains supplementary material available at 10.1186/s12913-021-07447-4.

## Background

Haemophilia A (HA) is a rare X-linked congenital bleeding disorder characterized by a deficiency of coagulation factor VIII which accounts for 80 to 85% of all haemophilia cases [1]. The level of baseline circulating factor VIII in these patients determines their tendency to bleed [1, 2]. Accordingly, while patients with mild HA (baseline level of circulating factor VIII 5 to 40 IU/dL or 5 to 40% of normal) rarely experience bleeding unless subjected to major trauma or surgery, patients with moderate (1 to 5 IU/dL or 1 to 5% of normal) or severe HA (< 1 IU/dL or < 1% of normal) more commonly experience spontaneous bleeding into joints, muscles, and internal organs throughout their lifetime [1, 2]. Data from the reference HA registry in Spain show that 21.3, 60.3%, and 66.1.3% of patients with mild, moderate and severe HA, respectively, experienced at least one annual bleeding episode in 2006, similar to values reported for moderate to severe HA in 2013 [3, 4]. Moreover, the reported annual frequency of bleeding episodes was 0.6, 3.2, and 5.4 in patients with mild, moderate and severe HA, respectively, similar to values reported for moderate to severe HA in 2013 [3, 4].

Regular administration of haemostatic agents, mainly factor VIII concentrate regular replacement therapy, is the standard approach to effectively prevent bleeding episodes (i.e., prophylaxis), specially joint haemorrhages that facilitate the development of arthropathy and disability [1, 5]. However, some patients with HA may develop a series of complications. Despite prophylaxis, many patients with HA continue to suffer from recurrent bleeding into joints and develop chronic arthropathy. Moreover, patients undergoing treatment may further develop inhibitors, alloantibodies to clotting factor VIII that make prevention and treatment of bleeding episodes difficult and further contribute to the development of arthropathy. Accordingly, haemophilia has been associated with impaired health-related quality of life (HRQoL) and a large economic burden [6, 7], further accentuated by increased severity [8, 9], the development of inhibitors [9–11], and the presence of arthropathy [5, 12], among others [7].

The development of inhibitors that neutralize the function of factor VIII concentrates, hampers the prevention and treatment of bleeding episodes [2]. Accordingly, these patients present significantly greater annual bleeding rates and a higher proportion suffer major bleeding episodes compared to patients without inhibitors [13]. In turn, patients with haemophilia that develop inhibitors present a worse HRQoL than patients without inhibitors [10]. Moreover, the presence of inhibitors has been associated with an increase in healthcare resource utilisation, direct healthcare costs, and indirect costs associated with the loss of work productivity for patients and informal caregivers compared to patients without inhibitors [9, 11].

Hemarthroses, or bleeding into joints, account for 70 to 80% of all bleeding episodes [1]. Acute hemarthroses result in acute synovitis which, if not appropriately managed, may develop into chronic synovitis and ultimately into chronic arthropathy [1]. Patients with severe HA and at least one joint with chronic synovitis have a significantly lower HRQoL (EQ-5D utility index of 0.73) than patients without chronic synovitis (EQ-5D utility index of 0.77) [5]. Similarly, a greater proportion of patients with chronic synovitis have reported problems associated with mobility, self-care, usual activities, pain/discomfort, and anxiety/depression compared to patients without chronic synovitis [5]. Moreover, the presence of chronic synovitis has been associated with an increase in healthcare resource utilisation and non-drug-related direct costs, compared to patients without chronic synovitis [12]. In conclusion, the development of complications in the management of HA (e.g. the development of arthropathy and/or inhibitors) has an impact on HRQoL and poses a large economic burden on the healthcare system, patients and caregivers, and society at large [7].

A recent longitudinal study showed that primary prophylaxis, that which is started before the onset of joint disease, the second clinically evident joint bleed, and 3 years of age, may preserve joint health and overall function in patients with HA [14]. Moreover, a recent systematic review showed that long-term prophylaxis for patients with HA results in fewer bleeding rates, reduced signs of arthropathy, better HRQoL, and greater labour productivity compared to the episodic treatment of bleeding episodes [15]. Data from the reference HA registry in Spain show that 65.6% of patients with moderate to severe HA were being treated with prophylaxis in 2013, 32.4% of which were under primary prophylaxis, which represents an important improvement with respect to data from 2006 [3, 4]. Unfortunately, 51.2% of patients with moderate to severe HA in Spain still developed established haemophilic arthropathy [3, 4].

To deliver ideal care, HA management within the Spanish National Health System (SNHS) should align with the principles of care outlined by the World Federation of Haemophilia [1]. Though adherence to these principles has increased in western European countries, including Spain, there is still much room for improvement [4, 16–19]. The latest analysis shows that Spain is lacking a nationally coordinated haemophilia care program, prophylaxis treatment is only available for some patients, there are insufficient adult patients on prophylaxis treatment regimens, home delivery of treatment is unavailable, and some specialist services are only sometimes available (e.g., rheumatology, orthopaedics, physiotherapy, dentistry, social and psychological support, general surgery, and urology, among others) [18]. The European principles of care may provide a common foundation for multiple stakeholders to collaborate and make decisions on the allocation of resources for HA management within the SNHS to achieve the best standards possible [1]. Hence, inequalities between patients with HA could be addressed no matter where they live.

The Social Return on Investment (SROI) provides the ideal framework to assess the multidimensional impact that such decisions would have relative to the investment required to put them into practice [20–22]. Previous experiences using this method have been used to guide decision-making in multiple healthcare areas such as chronic conditions [23], disability [24], loneliness [25], substance abuse [26], surgery [27], oncology [28, 29], cardiology [30, 31], nephrology [32], neurology [33–36], dermatology [37], ageing [38–40], or maternity [41–43], among others [44–48]. To our knowledge, no other study to date has used the SROI method in HA. Thus, the aims of the present study were to establish a set of proposals to improve HA management within the SNHS and to estimate the impact its hypothetical implementation would generate from a clinical, healthcare, economic, and social perspective.

## Methods

The present study was developed and reported following the stages of the SROI framework, a method which has been previously described elsewhere [20]. Briefly, a forecast-type SROI analysis was carried out to estimate the impact of implementing a set of proposals to improve HA management within the SNHS over a one-year timeframe, in relation to the investment needed to implement them.

This analysis was based primarily on relevant information obtained from structured discussions with stakeholders, scientific literature related to HA, official statistics (mainly those published by the Spanish National Institute of Statistics and the Spanish Ministry of Health, Consumer Affairs, and Social Welfare), and the median rates for health services published in the official bulletins of the Spanish Autonomous Communities. Prices were updated to 2019 euros according to the corresponding Consumer Price Index. Assumptions were made only if strictly necessary when data points required for the SROI analysis were either missing or unclear (e.g., multiple sources with different data points). All assumptions were based on information published in the scientific literature and/or expert consensus, and were validated by the latter, supporting the reliability of the data.

Given the nature of this study, approval by an institutional review board or an ethical review board was not required. Nevertheless, study procedures were in accordance with the Declaration of Helsinki 1975/83.

### Stage 1. Establishing scope and identifying stakeholders

To determine a set of proposals to improve HA management within the SNHS a Project Advisory Committee (PAC) and a Multidisciplinary Expert Committee (MEC) were set up. The PAC, which comprised two haematologists with important background in HA and the president of the Spanish Federation of Haemophilia, was convened to determine the key areas for the allocation of specific proposals to improve HA management (patients with arthropathy, patients with inhibitors, paediatric patients, and general) and confirm the profile of stakeholders to be included in the MEC. The MEC, which comprised the members of the PAC as well as a hospital manager, a former deputy director of pharmacy, two hospital pharmacists, a rehabilitation physician, a physical therapist, a nurse, a social worker, a patient with HA, and an informal caregiver of a patient with HA, was convened to agree on a set of proposals to improve HA management within the key areas. To do so, the members of the MEC were divided into three groups representing three different perspectives (physicians, other healthcare professionals, and patients). Each group identified and accurately defined specific proposals within each key area, which were then shared and discussed with the rest of the groups. Thereafter, each member of the MEC rated each proposal regarding their ability to improve the current management of HA on a scale of 0 (not important) to 10 (very important). Overall, 15 proposals were selected among those with the highest score within each area: 20% from the area of patients with arthropathy, 20% from the area of patients with inhibitors, 20% from the area of paediatric patients, and 40% from the general area, as determined by the PAC.

For each proposal, a review of the scientific and grey literature was carried out to narrow down the scope to specific activities and to identify the stakeholders who might affect or be affected by such activities.

### Stage 2. Identifying and valuing resources: investment

The resources required to deliver the activities within each proposal were identified and associated with specific stakeholders. Moreover, the costs of these resources were quantified by multiplying the number of resources by their unit prices. Following the current convention on the SROI method, no financial value was given to the time patients and informal caregivers spend on activities as they are considered the main beneficiaries of such activities. Thereafter, the total investment was obtained by adding up the investments of individual proposals.

For every type of resource within each proposal:

$$Investment\left({\EUR}\right)= Resources(N)\times Unit\ price\left({\EUR}\right)$$

### Stage 3. Identifying and valuing outcomes

The outcomes of the activities required to implement each proposal were identified and associated with specific stakeholders. These were quantified by establishing an indicator of the amount of change that would occur and applying a financial value to such change. Outcomes may be positive or negative, intended or unintended, and tangible or intangible. The latter (i.e., those without a market value) required the use of financial proxies through contingent valuation methods.

In addition to data collected from the scientific literature, this analysis was further supported by information provided by members of the MEC, which individually rated the impact of each proposal on different aspects of the lives of patients and informal caregivers on a scale of 0 (no positive impact) to 10 (largest positive impact) through an online questionnaire, and an online Focus Group with HA patients and informal caregivers.

For every outcome within each proposal:

$$Outcome\left({\EUR}\right)=Indicator\;of\;the\;amount\;of\;change\;(N)\times Financial\;value\;or\;financial\;proxy\left({\EUR}\right)$$

### Stage 4. Establishing the impact

To determine the proportion of each outcome that could be ascribed to the activities within each proposal and avoid over claiming, outcomes were individually adjusted by removing deadweight (the proportion of the outcome that would have occurred even if the activity had not taken place), attribution (the proportion of the outcome that would be due to the contribution of other activities), and displacement (the proportion of the outcome that would displace other outcomes) from their total value. Drop-off did not apply to this analysis given the one-year timeframe. Thereafter, the total impact was obtained by adding up the impacts of individual proposals.

For every outcome within each proposal:

$$Impact\;(\EUR)=Outcome\;(\EUR)\;\times\;\lbrack100\%-Deadweight\;(\%)\rbrack\times\lbrack100\;\%-Attribution\;(\%)\rbrack\times\lbrack100\%\;-\;Displacement\;(\%)\rbrack.$$

### Stage 5. Calculating the SROI

To obtain the SROI ratio, the total net impact associated with the hypothetical implementation of the set of proposals to improve HA management within the SNHS was divided by the total investment required to implement them. A SROI ratio greater than 1 is considered positive, meaning that the total impact is greater than the total investment required.

For the complete set of proposals:

$$SROI\ ratio\left({\EUR}\right)=\frac{Total\ impact\;\left({\EUR}\right)}{Total\ investment\;\left({\EUR}\right)}$$

Finally, a sensitivity analysis was performed to determine the effect of varying the assumptions made for specific data-points required to estimate either the investment, outcomes or adjustment factors. This sensitivity analysis was performed by varying the assigned percentages used on certain assumptions. These variations were supported by scientific literature and the members of the PAC, and validated by the latter. The analysis considered three different scenarios, a reference case, a best-case scenario (using assumptions that would imply a lower investment and/or yield a greater outcome), and a worst-case scenario (using assumptions that would imply a higher investment and/or yield a lower outcome).

## Results

The MEC agreed on a set of 15 proposals to improve HA management within the SNHS (Table 1). Proposal 1 has been used to exemplify results within the different stages of the SROI method where appropriate. For the complete list of proposals identified by the MEC and their scores, a detailed analysis of each proposal including a breakdown of investment and impact, and the complete list of assumptions including worst and best-case scenarios used in the sensitivity analysis, refer to Online Resource 1.

**Table 1:** Proposals to improve HA management within the SNHS

Area: General	
1	Assistance from multidisciplinary teams with all the relevant professionals involved.
2	Hospital case management nurse.
3	Training for general practitioners on the management of age-related comorbidities.
4	Coordination between primary care health centres and Haemophilia Treatment Centres through access to medical records throughout the country.
5	Networking between small centres and reference centres, and between reference centres themselves.
6	Home-delivery of hospital medication.
Area: Patients with Arthropathy	
7	Protocol for the treatment of pain and training for professionals related to the management of the pathology.
8	Early diagnosis of arthropathy to adapt prophylaxis.
9	Protocol for a prompt referral between regions to perform orthopaedic surgery in reference centres.
Area: Patients with Inhibitors	
10	Creation of a national registry of patients.
11	Training for patients with inhibitors and their families on management and care in haemophilia.
12	Inhibitor eradication protocols.
Area: Paediatric patients	
13	Training for parents on the management of HA, provided in health centres, and the creation of patient groups.
14	Training for education professionals on caring for students with HA in schools.
15	Training for paediatric patients on treatment adherence.

Proposal 1 included routine visits to key members of multidisciplinary teams (haematologists, nurses, rehabilitation or physical therapists, and social workers) and, if needed, referrals to other healthcare professionals (orthopaedic surgeons, psychologists, dental health professionals, and/or dietetics and nutrition professionals) for patients with HA. The required investment to deliver these activities contemplated the cost of one annual visit (for adults with mild or moderate HA) or two annual visits (for adults with severe HA or paediatric patients regardless of severity) to key members of the multidisciplinary team for those patients with HA who were not receiving assistance from any of such healthcare professionals. Moreover, the investment also contemplated the cost of an annual visit to any one of the other healthcare professionals related to HA for those patients which needed such assistance but were not yet receiving it. As a result of these visits, the SNHS would improve the overall management and comprehensive care of patients with HA. Moreover, patients with HA and/or their informal caregivers who were not already satisfied with the SNHS would improve their satisfaction, an outcome sharing value with other outcomes on increased satisfaction with the SNHS due to the incorporation of a hospital case management nurse in every haemophilia treatment centre, the optimization of electronic health records, or the development of an intranet encompassing haemophilia treatment centres and comprehensive care centres. Furthermore, patients with HA who were not already receiving the assistance they needed would increase their overall quality of life associated with improved function, mental health and/or empowerment. Finally, informal caregivers would increase their burden having to accompany patients to additional consultations, which would be compensated by observations of a reduction in work absenteeism. Accordingly, this example accounts for the multidimensional impact of interventions in HA. Table 2 shows a detailed description of the components of the impact for Proposal 1.

**Table 2:** Breakdown of the impact for Proposal 1. Assistance from multidisciplinary teams with all the relevant professionals involved

Return	Indicator	Proxy	Deadweight	Attribution	Total
1.1. The satisfaction of adult HA patients concerning the SNHS would improve.	Number of adult HA patients who would improve their satisfaction with the SNHS: 1841 [49, 50].	Average annual premium per private insurance policyholder: €797.86 [51].	Percentage of adult patients with haemophilia seen annually by nursing staff: 46.00% [52].	Percentage of the impact ascribed to returns 1.1, 2.4, 4.3 and 5.5: 40.00%.	€475,805
1.2. The empowerment and quality of life of patients with HA would improve, thanks to access to social work services.	Number of patients with HA who would be more empowered, thanks to access to social work services: 1298 [49, 53].	Cost of five family therapy sessions: €441.51 [54].	Percentage of haemophilia patients seen annually by social work: 10.74% [52].	N/A	€511,343
1.3. Oral bleeding would be avoided in patients with HA, thanks to access to dental services.	Number of patients with HA in whom the appearance of dental caries would be avoided: 1298 [49, 53].	Total cost per person associated with the burden of dental disease: €171.96 [55].	Percentage of patients with HA who are already being cared for by a dentist: 50.00% [53].	N/A	€111,559
1.4. The function and quality of life of patients with HA and arthropathy would be improved, thanks to access to rehabilitation/physiotherapy and orthopaedic surgery services.	Total number of Quality-Adjusted Life Years (QALY) that would be maintained when attended by a rehabilitation/physiotherapy or orthopaedic surgery professional: 52 [8, 49, 53].	Incremental cost-effectiveness threshold per QALY: €21,000.00 [56].	Percentage of patients with HA and arthropathy who are already being cared for by an orthopaedic surgery professional: 50.00% [53].	N/A	€544,950
1.5 The function and quality of life of patients with HA and obesity or overweight would be improved, thanks to access to diet and nutrition services	Total number of QALYs that would be gained from being cared for by a dietetic and nutrition professional: 34 [5, 49, 53, 57].	Incremental cost-effectiveness threshold per QALY: €21,000.00 [56].	Percentage of HA patients with obesity or overweight who are already being cared for by a professional in dietetics and nutrition: 50.00% [53].	N/A	€355,600
1.6 The quality of life of patients with HA and mental health disorders would be improved, thanks to access to psychology services	Total number of QALYs that would be gained by being cared for by a professional in psychology: 92 [5, 49, 53, 58].	Incremental cost-effectiveness threshold per QALY: €21,000.00 [56].	Percentage of patients with haemophilia seen annually by psychology: 15.73% [52].	N/A	€1,627,800
1.7. The satisfaction of informal caregivers of patients with HA concerning the SNHS would improve.	Number of informal caregivers of patients with HA who would improve their satisfaction with the SNHS: 969 [49, 50, 53].	Average annual premium per private insurance policyholder: €797.86 [51].	Percentage of patients with haemophilia seen annually by nursing staff: 51.73% [52].	Percentage of the impact ascribed to returns 1.7, 2.6, 4.4 and 5.7: 40.00%.	€223,985
TOTAL RETURN €3,851,042					

N/A: not applicable. Note: For each outcome, the following calculation was used to determine the impact: = Indicator (N) x Financial value or proxy (€) x [100% - Deadweight (%)] x [100% - Attribution (%)] x [100% - Displacement (%)]

The implementation of the complete set of 15 proposals to improve HA management within the SNHS would require a total investment of €2,342,950.33. Proposal 1 on providing assistance from multidisciplinary teams would account for the largest proportion of the investment (40.21%), followed by Proposal 8 on early diagnosis of arthropathy (12.86%) (Table 3). Moreover, the total investment would have a social impact of €14,595,640.62. Proposal 12 on developing a protocol to eradicate inhibitors would yield the greatest proportion of the impact (34.09%), followed by Proposal 1 on providing assistance from multidisciplinary teams (26.38%), and Proposal 7 on developing a protocol for pain management and associated training for healthcare professionals related to HA (22.17%) (Table 3).

**Table 3:** Investment and impact, and proportion of the total for each proposal (€ 2019)

Proposal	Investment (€)	Impact (€)	Investment (%)	Impact (%)
1	942,179.83	3,851,041.99	40.21	26.38
2	203,234.03	399,162.35	8.67	2.73
3	53,529.81	89,904.22	2.28	0.62
4	24,810.89	101,560.26	1.06	0.70
5	32,624.08	78,852.06	1.39	0.54
6	139,681.95	1,207,285.41	5.96	8.27
7	102,965.81	3,235,558.66	4.39	22.17
8	301,218.88	313,169.78	12.86	2.15
9	50,400.00	19,583.68	2.15	0.13
10	186,512.32	0.00^a^	7.96	0.00^a^
11	43,151.50	60,480.57	1.84	0.41
12	36,936.00	4,976,367.41	1.58	34.09
13	66,199.50	137,224.82	2.83	0.94
14	43,357.07	88,781.61	1.85	0.61
15	116,148.66	36,667.82	4.96	0.25
Total	2,342,950.33	14,595,640.62	100	100

^a^ Outcomes were not quantified as this proposal was intended to aid the development of future interventions including those in the present study

The resulting SROI ratio was estimated at 6.23:1, meaning that for every euro invested in the hypothetical implementation of the complete set of 15 proposals as an integral intervention would yield a social return of €6.23. Regarding the typology of the social return, 54.29% (€3.38) was associated with intangible returns while 45.71% (€2.85) was associated with tangible returns. Finally, the results of the sensitivity analysis showed that the SROI ratio could vary between 3.37:1 in the worst-case scenario and 9.69:1 in the best-case scenario, which would increase the impact up to 55.5%.

## Discussion

The aims of the present study were to establish a set of proposals to improve HA management within the SNHS and to estimate the impact its hypothetical implementation would generate from a multidimensional perspective. Multiple stakeholders associated with HA agreed on 15 proposals covering key areas of HA management: patients with arthropathy, patients with inhibitors, paediatric patients, and general. Overall, these proposals acknowledged existing gaps in the current compliance of the SNHS with the principles of care outlined by the World Federation of Haemophilia in 2020 [1]. Accordingly, the implementation of specific actions within each proposal would allow the SNHS to better meet the global standards of haemophilia care and close the gap between evidence-based recommendations and routine clinical practice. Previously identified shortcomings concerning the national organization of care, access to treatment regimens, or access to specialist care services in Spain could be mitigated by the implementation of specific proposals and activities [18]. Among them, the creation of a national haemophilia coordination group, the optimization of electronic health records, an intranet encompassing haemophilia treatment centres and comprehensive care centres, and a national haemophilia patient registry would improve the national organization of care and reduce health inequalities. Moreover, bringing hospital medication to the patients’ home would improve access to prophylactic treatment regimens; and programming routine visits to all key members of multidisciplinary teams, including any required referrals, and the implementation of a nurse coordinator in every haemophilia treatment centre would improve access to specialist care services. Other proposals focused on haemophilia education for patients, informal caregivers, teachers, and primary care physicians; the development of protocols for pain management and inhibitor eradication; and programmed routine assessments for early diagnosis of arthropathy, all of which would further contribute to the optimization of HA management.

Using the SROI framework, this study estimated that every euro invested in the hypothetical implementation of the complete set of 15 proposals would yield a social return of €6.23 (€3.37 - €9.69) of both tangible and intangible nature in similar proportions. The proposals with the greatest impact relative to investment were those associated with the development of evidence-based protocols to eradicate inhibitors and manage pain in patients with HA, as the development of a clinical protocol would require a relatively low investment compared to the multidimensional impact that the ideal management of these major HA complications would have.

Patients with HA may develop inhibitors, alloantibodies to clotting factor VIII that hamper the prevention and treatment of bleeding episodes [2, 59]. The present study estimated that developing and communicating an updated evidence-based clinical protocol for eradicating inhibitors would increase the success rate of the immune tolerance induction therapy, further contributing to reduce direct costs for the SNHS associated with savings on the treatment of patients with severe HA and inhibitors [9]. Accordingly, the presence of inhibitors has been associated with significantly greater health resource utilization and direct costs compared to patients without inhibitors [9, 11, 13]. Moreover, the present study contemplated intangible returns for patients associated with improvements in joint health, mobility, pain, and activities of the daily living. Accordingly, the scientific literature reports a high impact of inhibitor development on HRQOL and improvements have been reported with inhibitor treatment [10]. Furthermore, a large proportion of patients with haemophilia present acute pain due to hemarthroses and chronic pain due to chronic arthropathy [60]. The present study estimated that developing and communicating an updated evidence-based clinical protocol on haemophilia-related pain management followed by specific training for healthcare professionals involved in HA management would have a large impact on HRQOL and reduce caregiver burden. Accordingly, the scientific literature reports a high impact of pain on HRQOL [8, 60]. Other proposals with a large impact relative to investment were home-delivery of hospital medication, followed by the implementation of a multidisciplinary care model, and the implementation of electronic health records. However, the results of individual proposals should not be considered separately, as the complete set of 15 proposals to improve HA management within the SNHS was intended as an integral intervention to be implemented as a whole. Unfortunately, comparing the overall results of the present study is not possible as this is the first to estimate the multidimensional impact of an integral approach to improve HA management.

The results of the present study may provide valuable information to guide decision-making associated with the HA management. Compared to traditional frameworks, the SROI incorporates a broader concept of value into the evaluation of healthcare interventions through engaging multiple stakeholders to account for all relevant perspectives, using financial proxies to account for complex outcomes which are difficult to monetize, and considering both positive and negative impacts [43]. Moreover, the chain of events going from the investment in specific activities to their impact can be easily followed for each stakeholder and proposal within the SROI framework [43]. Regarding stakeholders, patients and informal caregivers were included as stakeholders for both the identification and evaluation of proposals. Accordingly, their unmet needs were taken into account and any intangible aspects, such as improved satisfaction with the SNHS, were incorporated into the analysis which would have not been considered otherwise.

However, there are several limitations which should also be taken into account. A forecast-type SROI analysis with a one-year timeframe provides an estimate of the potential short-term impact of the hypothetical implementation of a set of 15 proposals. In contrast, an evaluative SROI analysis based on real-world evidence may provide a more robust foundation for decision-making. Nevertheless, the forecast-type SROI analysis has been recommended to identify relevant data to be included in the process of an evaluative SROI analysis [20, 61]. Moreover, within the SROI framework, a certain degree of bias associated with the configuration of the MEC, the financial proxies used to account for intangible returns, or the assumptions made for missing or unclear data points may be introduced. Despite these limitations, certain actions were performed to minimize bias. First, the members of the MEC were selected according to their background in HA, and their ability to faithfully represent the perspective of those stakeholders in the SNHS, patients, and caregivers. Second, the financial proxies and assumptions used were supported by the scientific literature and members of the PAC. Third, the sensitivity analysis showed that the SROI ratio was still positive even in the worst-case scenario. Finally, the results of SROI analysis depend to some extent on how the evaluation method was applied and the specific setting of the intervention, and should therefore be taken with caution. Given that estimates of investment and impact were associated with the hypothetical implementation of the set of proposals within the SNHS, generalizability of results to other settings is difficult. Nevertheless, despite vast differences between countries regarding the implementation of haemophilia care principles, western European countries have grown in parallel and share similar gaps [18, 19]. Therefore, while generalizing the results to other settings is not advised, these may inform enquiries on resource allocation for the management of HA in other western European countries.

## Conclusions

The present study established a comprehensive set of proposals to improve the current HA management within the SNHS, which may contribute to bridge the gap with the principles of care outlined by the World Federation of Haemophilia in 2020. Implemented as a whole, specific activities within proposals would yield an overall positive social impact. The results of the present study can be used to inform policy and practice within the SNHS such that interventions that may potentially improve current public health challenges associated with the management of HA may be implemented.

## Supplementary Information

**Additional file 1.**

## Data Availability

All data generated or analysed during this study are included in this published article [and its supplementary information files].

## Abbreviations

FEDHEMO: Federación Española de Hemofilia
HA: Haemophilia A
HRQoL: Health-related quality of life
MEC: Multidisciplinary Expert Committee
PAC: Project Advisory Committee
SNHS: Spanish National Health System
SROI: Social Return on Investment
