# Supplementary material for: The impact of improving haemophilia A management within the Spanish National Healthcare System: a social return on investment analysis
Source: BMC Health Serv Res. 2022 Jan 26;22:115. doi: 10.1186/s12913-021-07447-4 (PMC8793183; doi:10.1186/s12913-021-07447-4)
Supplement: Supplementary file 1 — Additional file 1. [file 12913_2021_7447_MOESM1_ESM.docx]

ONLINE RESOURCE 1

INDEX

[1. Abbreviations 2](#_Toc83315754)

[2. Initial list of proposals 3](#_Toc83315755)

[2.1 Area: general 4](#_Toc83315756)

[2.2 Area: patients with arthropathy 6](#_Toc83315757)

[2.3 Area: patients with inhibitors 8](#_Toc83315758)

[2.4 Area: pediatric patients 10](#_Toc83315759)

[3. Impact map and breakdown of investment and return 12](#_Toc83315760)

[3.1 Investment 12](#_Toc83315761)

[3.2 Return 27](#_Toc83315762)

[4. List of assumptions 53](#_Toc83315763)

[4. Table Index 70](#_Toc83315764)

[5. References 73](#_Toc83315765)

# Abbreviations

| HA | Haemophilia A |
| --- | --- |
| ITI | Immune Tolerance Induction |
| NHS | National Health System |
| NHSEHR | National Health System Electronic Health Record |
| PHC | Primary Health Care |
| QALY | Quality Adjusted Life Years |
| SROI | Social Return on Investment |
| SC | Specialised Care |

# Initial list of proposals

Below is the initial list of proposals for each area, as well as an average assessment of their importance carried out by the members of the Multidisciplinary Working Group. As indicated in the methodology, according to the criteria agreed with the Advisory Committee, 40% of the most voted proposals in the General Area were selected, 20% of the most voted proposals in the Area of Patients with Arthropathy, 20% of the most voted proposals in the Area of Inhibitor Patients and 20% of the most voted proposals in the Area of Paediatric Patients.

## Area: general

The initial list of proposals for the general area is set out below, according to its original wording. Final proposals that are part of the SROI analysis are shaded in blue.

Table 1. Proposals for improving the approach to Haemophilia A (HA). General area.

| Proposals^[[1]](#footnote-1)^ | Average Rating* |
| --- | --- |
| Multidisciplinary team: haematologist, orthopaedic surgery, rehabilitation, physiotherapist, pharmacist, nursing, social worker, dietician, dentist, psychologist. | 9.15 |
| Nurse case manager. | 8.77 |
| To involve the primary care physician (PCP) in the management of ageing-related comorbidities: arterial hypertension, diabetes, hyperlipidaemia, osteoporosis, depression, by identifying training needs by the MAP (online survey proposed by scientific societies). | 8.69 |
| Better coordination between primary care health centres and haemophilia units through nationwide access to medical records, including hospitals and specialist care centres. | 8.46 |
| To consolidate a network (application/forum) between smaller and bigger and reference centres. | 8.23 |
| More accessible home medication. | 8.23 |
| To train PC professionals in haemophilia: doctor, nurse, dentist through face-to-face courses taught by an expert haematologist and patient. | 8.15 |
| To better inform the patient during the medical appointment about the MAP, telling him about his comorbidities and the advantages of having it controlled in his health centre. | 8.15 |
| The PC nurse should administer the substitution treatment of the patient with congenital coagulopathies. | 8.15 |
| To standardize training and support programs through a multidisciplinary team before the diagnosis: psychologist, rehabilitator, haematologist, traumatologist, nursing. | 8.00 |
| To guarantee the continuity of access to the drug once the clinical trial is finished, if the result has been positive. | 7.92 |
| Face-to-face education in patient’s appointment by all team: adherence, administration schedules. | 7.85 |
| Clinical pathway in haemophilia. | 7.85 |
| Platform with educational material by age group and subject. | 7.77 |
| Annual multidisciplinary group training (hospital setting) | 7.77 |
| Establishment of haematologist-MAP telematic communication. | 7.77 |
| Multidisciplinary reference centre in each autonomous community. | 7.69 |
| To improve the education of mild patients with audio-visual material and telematic support: mobile messaging, precautions reminder, etc. | 7.50 |
| To carry out a comparative study of the cost of patients with different health itineraries for the NHS. | 7.46 |
| To agree on the information to be delivered by the team. | 7.38 |
| To standardize treatment at a national level through a health technology assessment agency guide, in coordination with the Royal Victoria Eugenia Foundation. | 7.08 |
| To raise social awareness about haemophilia: world haemophilia day, programs in educational centres, health centres. | 6.77 |
| Curricular adaptation in patients with high school absenteeism by the school's guidance team: development of online classes, flexibility in the evaluation, etc. | 6.77 |
| Publicize the complementary portfolios of the most advanced Autonomous Communities in the approach to haemophilia through an informative dossier. | 6.38 |
| Provide all haemophilia units with vein finders. | 5.92 |
| Periodic reminders of corporate activities for all patients with coagulopathies (e.g.: world haemophilia day ...). | 5.85 |
| Provide all patients with vein finders. | 5.54 |

* 0 = not at all important; 10 = extremely important.

## Area: patients with arthropathy

The initial list of proposals for the area of patients with arthropathy is set out below, according to their original wording. Final proposals that are part of the SROI analysis are shaded in blue.

Table 2. Proposals for improving the approach to HA. Area of patients with arthropathy.

| Proposals^[[2]](#footnote-2)^ | Average Rating* |
| --- | --- |
| Pain treatment. To improve the training of healthcare professionals in pain management. Courses, training in pain unit. Protocols | 9.54 |
| Early diagnosis of arthropathy to adapt prophylaxis. | 9.54 |
| Establish a rapid referral protocol between Autonomous Communities for orthopaedic surgery (from synoviorthesis to prosthesis). | 9.31 |
| Access to orthopaedic surgery in Reference Centres and/or centres with experience. | 9.17 |
| Multidisciplinary approach. Specialized musculoskeletal team (rehabilitation, orthopaedic surgery and traumatology, physiotherapy). | 9.15 |
| Arthrocentesis in acute hemarthroses. | 8.82 |
| Training material (videos and workshops) for patients/parents on sequelae in haemophilia (hemarthrosis, synovitis, arthropathy) (with a previous deliverable, a brochure that leads you to the videos, a link ...). | 8.77 |
| Train patients in analgesia/anti-inflammatories. | 8.69 |
| Individualized counselling for the transition from on-demand treatment to prophylaxis. | 8.62 |
| Access to specialized physiotherapy | 8.58 |
| Preparation of a consensus guide for the administration of analgesic drugs (in acute and chronic pain). | 8.54 |
| Coordinate the entire healthcare process. Case managers in haemophilia units. | 8.50 |
| Unify access to technical aids and complete financial coverage (access for all). | 8.46 |
| Programs and recommendations for adapted and recreational physical activity. Written and audio-visual information from rehabilitation. | 8.31 |
| Make a biannual joint follow-up protocol in patients with arthropathy and on-demand; make annual joint follow-up protocol in patients with arthropathy in prophylaxis. | 8.00 |
| Facilitate access to Pain Units. | 7.77 |
| Grade of disability. Reports of functional status. | 7.77 |
| Architectural barriers. Elimination, reduction, signalling ... | 7.67 |
| Coordination with community centres or community gyms with maintenance programs. | 7.62 |
| Follow-up with ultrasound at the point of care. | 7.54 |
| Social workers at the hospital as supporters; giving information, facilitation, resources, help. | 7.23 |
| To train a healthcare professional in pain care, so he can do the follow up with patients (the person would depend on the structure of the hospital) | 7.23 |
| Arthropathy self-assessment app with frequently asked questions that connects with the rehabilitator. | 6.75 |

* 0 = not at all important; 10 = extremely important.

## Area: patients with inhibitors

The initial list of proposals for the area of patients with inhibitors is set out below, according to its original wording. Final proposals that are part of the SROI analysis are shaded in blue.

Table 3. Proposals for improving the approach to HA. Area of patients with inhibitors.

| Proposals^[[3]](#footnote-3)^ | Average Rating* |
| --- | --- |
| National registry of patients (including carriers) and inhibitors. | 9.31 |
| Training for patients and their families in the management and care of haemophilia (by the entire multidisciplinary team). | 9.23 |
| To establish inhibitor eradication protocols. | 8.69 |
| Updating of the consensus document of the Royal Victoria Eugenia Foundation on prophylactic, on-demand and surgical treatment of patients with inhibitors, with the inclusion of new treatments. | 8.67 |
| Establish a protocol for direct access to referral centres for surgical interventions for patients from other regions or provinces. | 8.62 |
| To inform patients about access to currently available therapeutic alternatives, through the distribution of informative material to patients and hospitals. | 8.46 |
| The Ministry of Health must agree on a price with pharmaceutical companies that allows access to approved treatments for all haemophiliacs with an inhibitor (emicizumab etc.). | 8.38 |
| The haematologist specifies and decides which patients are eligible for Immune Tolerance Induction (ITI), for how long and with which product (plasma/recombinant). | 8.31 |
| Economic and clinical study of the treatment comparing: emicizumab +/- ITI. | 8.08 |
| To prepare a protocol for clinical suspicion and early diagnosis aimed for professionals in contact with the patient. | 8.00 |
| To establish specific psychosocial support programs (for example, weekly, for about 6 months). | 7.85 |
| Increase the number of musculoskeletal examinations in appointments of patients with inhibitor and physical treatments, led by experts. | 7.85 |
| Study of needs through a survey to detect current needs, both of patients and caregivers. | 7.77 |
| Psychological approach for caregivers to avoid overprotection. | 7.77 |
| To study the efficacy of ITIs through a prospective national study. | 7.69 |
| Determine which laboratory tests are more suitable for monitoring the effect of new molecules. | 7.69 |
| Preparation of a consensus guide of etiological factors for the detection of the inhibitor by the team. | 7.54 |
| Helpline for patients and caregivers. | 7.15 |
| To identify the product that the patient was receiving at the time the inhibitor was detected (research work). | 6.85 |

* 0 = not at all important; 10 = extremely important

## Area: paediatric patients

Below is the initial list of proposals for the area of paediatric patients, according to its original wording. Final proposals that are part of the SROI analysis are shaded in blue.

Table 4. Proposals for improving the approach to HA. Area of paediatric patients.

| Proposals^[[4]](#footnote-4)^ | Average Rating* |
| --- | --- |
| Establish specific training programs for parents in the management of the disease, to avoid overprotection and to deliver up to date information on the approach to haemophilia /// To promote an active life and psychomotor development of children, in health centres and patient groups. | 9.38 |
| Training on the care of students with haemophilia. Training guides for the management of acute bleeding and ways of collaboration with schools. | 9.00 |
| To educate on the consequences of lack of treatment adherence, through professionals, patient societies, expert patients (understand significance). Intensify educational programs in preadolescence. | 9.00 |
| Control by expert haematologists and/or paediatricians specialized in haemophilia, in coordination with the main paediatrician. | 8.92 |
| To implement programs of self-treatment and care and management of the reservoir in patients and their families in all centres. | 8.92 |
| Establish a protocol for transfer to reference centres for haemophilia, in cases of clinical suspicion or diagnosis, through the scientific societies of Neonatology and Paediatrics. | 8.77 |
| Advise on healthy lifestyles from an early age, including physical activity, diet, sedentary lifestyle. Professionals, groups, through teaching materials. | 8.62 |
| To include an alert in health cards that informs if the patient has haemophilia. | 8.46 |
| Protocolize the start times of prophylactic treatments. | 8.23 |
| To identify the work needs of parents of children with haemophilia and facilitate work and hospital reconciliation. /// Family and work conciliation, expand social measures in this sense. | 8.15 |
| Frequent joint monitoring with sensitive methods for early diagnosis of joint changes that allow assessing the efficacy of prophylaxis. | 8.15 |
| To implement psychological support programs for parents of children with haemophilia. | 8.15 |
| Bring home care closer and reduce hospital care (home nursing for self-administration, exercise as leisure). | 8.00 |
| Implementation of strategies to avoid the development of inhibitors, unifying criteria. A consensus of experts through scientific societies. | 7.85 |
| Raise awareness about haemophilia in schools to avoid exclusion. | 7.77 |
| To prepare audio-visual material for caregivers so that they learn how to detect bleeding problems. | 7.62 |
| Deliver care in friendly and child-friendly environments (including all care and waiting spaces). | 7.38 |
| To train professionals in painless puncture (distraction, anaesthetic cream ...) | 7.38 |
| Promote App development, gamification and new technologies that favour self-care. | 6.92 |
| Provide the Haematology Services with vaccines, since intramuscular vaccines for patients with haemophilia require specific management. | 6.54 |
| To facilitate access to joint and cranial protection material for children with haemophilia who need it. | 6.46 |

* 0 = not at all important; 10 = extremely important

# Impact map and breakdown of investment and return

## Investment

The following tables detail the investment for each proposal.

Table 5. Proposal 1 Investment Breakdown: Attention by multidisciplinary teams with all specialities involved.

| Interest groups | Investment description | Investment breakdown | |
| --- | --- | --- | --- |
| Nursing professionals | Working time | Number of paediatric HA patients (<14 years) ^[1]^. | 479 |
|  |  | Percentage of paediatric patients with haemophilia seen annually by nursing staff ^[2]^. | 77.00% |
|  |  | Number of adult HA patients (≥ 14 years) ^[1]^. | 2,116 |
|  |  | Percentage of adult patients with severe HA ^[1]^. | 30.63% |
|  |  | Percentage of adult patients with haemophilia seen annually by nursing staff ^[2]^. | 46.00% |
|  |  | Number of annual check-ups for adult patients with severe HA and paediatric patients ^[3]^. | 2 |
|  |  | Percentage of adult patients with mild HA ^[1]^. | 55.88% |
|  |  | Percentage of adult patients with moderate HA ^[1]^. | 13.49% |
|  |  | Number of annual check-ups for adult patients with mild-moderate HA ^[3]^. | 1 |
|  |  | Cost of hospital nursing visits ^[4]^. | €42.00 |
|  |  | Total (€) | €71,940.23 |
| Rehabilitation / Physical Therapy Professionals | Working time | Number of paediatric HA patients (<14 years) ^[1]^. | 479 |
|  |  | Percentage of paediatric patients with haemophilia seen annually for rehabilitation ^[2]^. | 64.00% |
|  |  | Number of adult HA patients (≥ 14 years) ^[1]^. | 2,116 |
|  |  | Percentage of adult patients with severe HA ^[1]^. | 30.63% |
|  |  | Percentage of adult patients with haemophilia seen annually for rehabilitation ^[2]^. | 44.00% |
|  |  | Number of annual check-ups for adult patients with severe HA and paediatric patients ^[3]^. | 2 |
|  |  | Percentage of adult patients with mild HA ^[1]^. | 55.88% |
|  |  | Percentage of adult patients with moderate HA ^[1]^. | 13.49% |
|  |  | Number of annual check-ups for adult patients with mild-moderate HA ^[3]^. | 1 |
|  |  | Cost of the first visit to a specialist ^[4]^. | €151.75 |
|  |  | Cost of successive visit to a specialist ^[4]^. | €88.30 |
|  |  | Total (€) | €253,255.96 |
| Social Workers | Working time | Number of paediatric HA patients (<14 years) ^[1]^. | 479 |
|  |  | Percentage of paediatric patients with haemophilia seen annually by social work ^[2]^. | 14.00% |
|  |  | Number of adult HA patients (≥ 14 years) ^[1]^. | 2,116 |
|  |  | Percentage of adult patients with severe HA ^[1]^. | 30.63% |
|  |  | Percentage of adult patients with haemophilia seen annually by social work ^[2]^. | 10.00% |
|  |  | Number of annual check-ups for adult patients with severe HA and paediatric patients^[3]^. | 2 |
|  |  | Percentage of adult patients with mild HA ^[1]^. | 55.88% |
|  |  | Percentage of adult patients with moderate HA ^[1]^. | 13.49% |
|  |  | Number of annual check-ups for adult patients with mild-moderate HA ^[3]^. | 1 |
|  |  | Cost of visit to social work ^[4]^. | €36.27 |
|  |  | Total (€) | €120,118.58 |
| Orthopaedic Surgery Professionals | Working time | Number of patients with HA ^[1]^. | 2,595 |
|  |  | Percentage of patients with HA and at least one joint with signs of arthropathy ^[5]^. | 56.90% |
|  |  | Percentage of patients with HA and arthropathy who are already being cared for by an orthopaedic surgery professional ^[6]^. | 50.00% |
|  |  | Cost of the first visit to a specialist ^[4]^. | €151.75 |
|  |  | Total (€) | €112,033.87 |
| Psychology professionals | Working time | Number of adult HA patients (≥ 14 years) ^[1]^. | 2,116 |
|  |  | Number of paediatric HA patients (<14 y ears) ^[1]^. | 479 |
|  |  | Percentage of haemophilia patients with symptoms of depression (used as a proxy for mental health disorders) ^[7]^. | 41.70% |
|  |  | Percentage of adult patients with haemophilia seen annually by psychology ^[2]^. | 10.00% |
|  |  | Percentage of paediatric patients with haemophilia seen annually by psychology ^[2]^. | 41.00% |
|  |  | Cost of the first visit to a specialist ^[4]^. | €151.75 |
|  |  | Total (€) | €102,280.39 |
| Dental professionals | Working time | Number of patients with HA^[1]^. | 2,595 |
|  |  | Percentage of patients with HA who are already being cared for by a dentist ^[6]^. | 50.00% |
|  |  | Cost of the first visit to a specialist ^[4]^. | €151.75 |
|  |  | Total (€) | €196,896.08 |
| Dietetics and nutrition professionals | Working time | Number of adult HA patients (≥ 14 years) ^[1]^. | 2,116 |
|  |  | Percentage of adults with haemophilia who are obese or overweight ^[8]^. | 49.10% |
|  |  | Number of paediatric HA patients (<14 years) ^[1]^. | 479 |
|  |  | Percentage of paediatric haemophilia patients who are obese or overweight ^[8]^. | 18.80% |
|  |  | Percentage of patients with HA and obesity or overweight who are already being cared for by a professional in dietetics and nutrition ^[6]^. | 50.00% |
|  |  | Cost of the first visit to a specialist ^[4]^. | €151.75 |
|  |  | Total (€) | €85,654.73 |
| PROPOSAL 1 TOTAL INVESTMENT | | | €942,179.83 |

Table 6. Proposal 2 Investment Breakdown: Nursing hospital case manager.

| Interest groups | Investment description | Investment breakdown | |
| --- | --- | --- | --- |
| National Health System | Economic resources | Cost of nursing training in haemophilia ^[9]^. | €3,983.89 |
|  |  | Number of Haemophilia Treatment Centres (HTC)^[10]^. | 64 |
|  |  | Percentage of nurses with extensive experience in haemophilia (used as a proxy for the level of implantation of the nurse case manager)^[11]^. | 44.00% |
|  |  | Total (€) | €142,782.62 |
| Nursing hospital case manager | Working time | Number of adult HA patients (≥ 14 years)^[1]^. | 2,116 |
|  |  | Percentage of adult patients with mild HA^[1]^. | 55.88% |
|  |  | Percentage of adult patients with moderate HA^[1]^. | 13.49% |
|  |  | Number of telephone calls per year per patient for the follow-up of patients with mild-moderate HA ^[6]^. | 2 |
|  |  | Percentage of adult patients with severe HA^[1]^. | 30.63% |
|  |  | Number of paediatric HA patients (<14 years)^[1]^. | 479 |
|  |  | Number of telephone calls per year per patient for the follow-up of patients with severe and paediatric HA. ^[6]^. | 4 |
|  |  | Percentage of nurses with extensive experience in haemophilia (used as a proxy for the level of implantation of the nurse case manager)^[11]^. | 44.00% |
|  |  | Telephone appointment cost ^[4]^. | 14.50 € |
|  |  | Total (€) | €60,451.41 |
| PROPOSAL 2 TOTAL INVESTMENT | | | €203,234.03 |

Table 7. Proposal 3 Investment Breakdown: Training for Primary Care Medicine Professionals on the Management of Comorbidities Related to Aging.

| Interest groups | Investment description | Investment breakdown | |
| --- | --- | --- | --- |
| National Health System | Economic resources | Average cost of an online survey aimed at PC professionals ^[12]^. | €15,000 |
|  |  | Total (€) | €15,000.00 |
| National Health System | Economic resources | Number of HTC^[10]^. | 64 |
|  |  | Number of face-to-face sessions per year by HTC, for the training of PC medicine professionals in the management of ageing-related comorbidities ^[6]^. | 1 |
|  |  | Cost of a first application for the accreditation of a face-to-face course ^[13]^. | €102.01 |
|  |  | Cost of successive editions for the accreditation of a face-to-face course ^[13]^. | €30.60 |
|  |  | Cost of scientific endorsement from the Spanish Society of Haematology and Hemotherapy ^[14]^. | €1,500.00 |
|  |  | Cost of sponsorship from the Spanish Society of Thrombosis and Haemostasis to scientific activities or publications developed by other Scientific Societies ^[15]^. | €0.00 |
|  |  | Cost of scientific endorsement from the Spanish Society of Primary Care Physicians ^[16]^. | €3,000.00 |
|  |  | Total (€) | €6,529.81 |
| Haematology Professionals | Working time spent on training outside of working hours. | Number of HTC^[10]^. | 64 |
|  |  | Number of face-to-face sessions per year by HTC, for the training of PC medicine professionals in the management of ageing-related comorbidities ^[6]^. | 1 |
|  |  | Duration (hours) of face-to-face sessions for the training of PC medicine professionals ^[6]^. | 4 |
|  |  | Cost per hour of face-to-face training ^[17]^. | €125.00 |
|  |  | Total (€) | €32,000.00 |
| PROPOSAL 3 TOTAL INVESTMENT | | | €53,529.81 |

Table 8. Proposal 4 Investment Breakdown: Coordination between primary care health centres and haemophilia units through access to medical records throughout the national territory.

| Interest groups | Investment description | Investment breakdown | |
| --- | --- | --- | --- |
| National Health System | Economic resources | Number of patients with HA^[1]^. | 2,595 |
|  |  | Expenditure per person in vertical information systems per 100,000 people of the protected population ^[18]^. | €4.57 |
|  |  | Total (€) | €11,858.89 |
| National Health System | Economic resources | Cost of an online course for the use of the National Health System Digital Medical Record ^[12]^. | €12,952.00 |
|  |  | Total (€) | €12,952.00 |
| PROPOSAL 4 TOTAL INVESTMENT | | | €24.810.89 |

Table 9. Proposal 5 Investment Breakdown: Networking between small and reference centres, and between reference centres with each other.

| Interest groups | Investment description | Investment breakdown | |
| --- | --- | --- | --- |
| National Health System | Economic resources | Intranet software cost ^[12]^. | €10,222.08 |
|  |  | Web design cost ^[12]^. | €6,250.00 |
|  |  | Total (€) | €16,472.08 |
| National Health System | Economic resources | Cost of an online training course for managing the intranet ^[12]^. | €12,952.00 |
|  |  | Total (€) | €12,952.00 |
| Health professionals linked to HA management | Working time | Number of annual posts on the intranet blog for networking ^[6]^. | 4 |
|  |  | Fees for each blog post on the intranet for networking ^[12]^. | €800.00 |
|  |  | Total (€) | €3,200.00 |
| PROPOSAL 5 TOTAL INVESTMENT | | | €32,624.08 |

Table 10. Proposal 6 Investment Breakdown: Bringing hospital medication closer to patients.

| Interest groups | Investment description | Investment breakdown | |
| --- | --- | --- | --- |
| National Health System | Economic resources | Number of paediatric HA patients (<14 years) ^[1]^. | 479 |
|  |  | Percentage of paediatric patients with moderate HA ^[1]^. | 13.56% |
|  |  | Percentage of moderate paediatric patients receiving prophylaxis treatment ^[19]^. | 43.33% |
|  |  | Percentage of paediatric patients with severe HA ^[1]^. | 41.53% |
|  |  | Percentage of paediatric patients with severe HA receiving prophylaxis treatment^[19]^. | 71.50% |
|  |  | Number of adult HA patients (≥ 14 years) ^[1]^. | 2,116 |
|  |  | Percentage of adult patients with moderate HA ^[1]^. | 13.49% |
|  |  | Percentage of moderate adult patients receiving prophylaxis treatment ^[19]^. | 16.96% |
|  |  | Percentage of adult patients with severe HA ^[1]^. | 30.63% |
|  |  | Percentage of seriously ill adult patients receiving prophylaxis treatment ^[19]^. | 34.74% |
|  |  | Number of patients who already benefit from the home medication transport service ^[20,21]^. | 70 |
|  |  | Average cost of the Home Delivery project (transport of medication to home in haemophilia) per patient ^[20]^. | € 373.50 |
| PROPOSAL 6 TOTAL INVESTMENT | | | €139,681.95 |

Table 11. Proposal 7 Investment Breakdown: Protocol for pain treatment and training for professionals related to the pathology.

| Interest groups | Investment description | Investment breakdown | |
| --- | --- | --- | --- |
| National Health System | Economic resources | Average cost of preparing and publishing a scientific article in a scientific journal ^[12]^. | €8,000.00 |
|  |  | Average cost of preparing and presenting (poster or oral communication) in a scientific congress ^[12,22]^. | €3,500.00 |
|  |  | Cost of scientific congress registration fee ^[12]^. | €636.00 |
|  |  | Accommodation cost to attend a scientific congress ^[12]^. | €800.00 |
|  |  | Total (€) | €12,936.00 |
| National Health System | Economic resources | Number of HTC ^[10]^. | 64 |
|  |  | Number of sessions per HTC of continuous face-to-face training per year in HA, aimed at healthcare professionals in pain units ^[6]^. | 1 |
|  |  | Cost of a first application for the accreditation of a face-to-face course ^[13]^. | €102.01 |
|  |  | Cost of following editions for the accreditation of a face-to-face course ^[13]^. | €30.60 |
|  |  | Total (€) | €2,029.81 |
| Development of a pain treatment protocol through a working group | Working time | Number of experts who would participate in the Working Group to develop the protocol ^[23]^. | 10 |
|  |  | Fees/expert for the development of the protocol ^[12]^. | €2,400.00 |
|  |  | Total (€) | €24,000.00 |
| Professionals of pain units | Working time spent on training outside of working hours | Number of HTC ^[10]^. | 64 |
|  |  | Number of sessions per HTC of continuous face-to-face training per year in HA, aimed at pain units healthcare professionals ^[6]^. | 1 |
|  |  | Duration (hours) of training for healthcare professionals in HA pain units (2 days, 4 hours a day) ^[6]^. | 8 |
|  |  | Cost per hour of face-to-face training ^[17]^. | €125.00 |
|  |  | Total (€) | €64,000.00 |
| PROPOSAL 7 TOTAL INVESTMENT | | | €102,965.81 |

Table 12. Proposal 8 Investment Breakdown: Early Diagnosis of Arthropathy to Adapt Prophylaxis.

| Interest groups | Investment description | Investment breakdown | |
| --- | --- | --- | --- |
| National Health System | Economic resources | Number of paediatric HA patients (<14 years) ^[1]^. | 479 |
|  |  | Number of adult HA patients (≥ 14 years) ^[1]^. | 2,116 |
|  |  | Percentage of adult patients with severe HA ^[1]^. | 30.63% |
|  |  | Number of annual check-ups for adult patients with severe HA and paediatric patients ^[3]^. | 2 |
|  |  | Percentage of adult patients with mild HA ^[1]^. | 55.88% |
|  |  | Percentage of adult patients with moderate HA ^[1]^. | 13.49% |
|  |  | Number of annual check-ups for adult patients with mild-moderate HA ^[3]^. | 1 |
|  |  | Joint ultrasound cost ^[4]^. | €80.92 |
| PROPOSAL 8 TOTAL INVESTMENT | | | €301,218.88 |

Table 13. Proposal 9 Investment Breakdown: Rapid referral protocol between autonomous communities to perform orthopaedic surgeries in reference centres.

| Interest groups | Investment description | Investment breakdown | |
| --- | --- | --- | --- |
| National Health System | Economic resources | Number of members of the National Haemophilia Coordination Group ^[6]^. | 21 |
|  |  | Fees/member of the National Haemophilia Coordination Group ^[12]^. | €2,400.00 |
| PROPOSAL 9 TOTAL INVESTMENT | | | €50,400.00 |

Table 14. Proposal 10 Investment Breakdown: Development of a patient national registry.

| Interest groups | Investment description | Investment breakdown | |
| --- | --- | --- | --- |
| National Health System | Economic resources | Database software cost ^[12]^. | €3,862.32 |
|  |  | Web design cost ^[12]^. | €6,250.00 |
|  |  | Cost of scientific endorsement from the Spanish Society of Haematology and Hemotherapy ^[14]^. | €1,500.00 |
|  |  | Total (€) | €11,612.32 |
| Multidisciplinary committee of experts | Working time | Number of experts needed for the multidisciplinary expert committee of the national registry ^[6]^. | 5 |
|  |  | Fees/expert for the development of a national data registry of patients with HA ^[12]^. | €2,400.00 |
|  |  | The average cost of preparing and publishing a scientific article in a scientific journal ^[12]^. | €8,000.00 |
|  |  | Total (€) | €20,000.00 |
| Coordination group | Working time | Number of members of the national registry coordination group ^[6]^. | 21 |
|  |  | Fees/member of the national registry coordination group ^[12]^. | €2,400.00 |
|  |  | Total (€) | €50,400.00 |
| Clinical Research Organization | Working time | Fees / Clinical Research Organization for the national registry data management ^[12]^. | €85,500.00 |
|  |  | Total (€) | €85,500.00 |
| Associations of patients with HA | Human resources | Required number of lectures on the importance of the national register ^[6]^. | 19 |
|  |  | Cost of fees plus information logistics per association ^[12]^. | €1,000.00 |
|  |  | Total (€) | €19,000.00 |
| PROPOSAL 10 TOTAL INVESTMENT | | | €186,512.32 |

Table 15. Proposal 11 Investment Breakdown: Training for patients with inhibitor, and their families, on the management and care of haemophilia.

| Interest groups | Investment description | Investment breakdown | |
| --- | --- | --- | --- |
| National Health System | Economic resources | Informative poster design cost ^[12]^. | €2,000.00 |
|  |  | Number of HTC ^[10]^. | 64 |
|  |  | Number of informative posters ^[6]^. | 2 |
|  |  | Printing cost for each informative poster ^[12]^. | €10.00 |
|  |  | Cost of postage of a package ^[12]^. | €8.00 |
|  |  | Cost of scientific endorsement from the Spanish Society of Haematology and Hemotherapy ^[14]^. | €1,500.00 |
|  |  | Cost of sponsorship from the Spanish Society of Thrombosis and Haemostasis to scientific activities or publications developed by the Pharmaceutical Industry ^[15]^. | €907.50 |
|  |  | Total (€) | €6,199.50 |
| National Health System | Economic resources | Cost of an online course aimed at patients with prevalent HA and inhibitor ^[12]^. | €12,952.00 |
|  |  | Total (€) | €12,952.00 |
| Multidisciplinary team: haematology, nursing, surgery, rehabilitation, orthopaedics, psychology. | Working time spent on training outside of working hours. | Number of HTC^[10]^. | 64 |
|  |  | Number of workshops per year by HCT aimed at patients with incidental HA and inhibitor, and their families ^[6]^. | 1 |
|  |  | Length (hours) of the workshops aimed at patients with incidental HA and inhibitor, and their families ^[6]^. | 3 |
|  |  | Cost per hour of face-to-face training ^[17]^. | €125.00 |
|  |  | Total (€) | €24,000.00 |
| PROPOSAL 11 TOTAL INVESTMENT | | | €43,151.50 |

Table 16. Proposal 12 Investment Breakdown: Inhibitor eradication protocols.

| Interest groups | Investment description | Investment breakdown | |
| --- | --- | --- | --- |
| National Health System | Economic resources | Average cost of preparing and publishing a scientific article in a scientific journal ^[12]^. | €8,000.00 |
|  |  | Average cost of preparation and presentation (poster or oral communication) in a scientific conference ^[12,22]^. | €3,500.00 |
|  |  | Scientific congress registration fee ^[12]^. | €636.00 |
|  |  | Accommodation cost to attend a scientific congress ^[12]^. | €800.00 |
|  |  | Total (€) | €12,936.00 |
| Development of the inhibitor eradication protocol through a working group | Working time | Number of experts who would participate in the protocol development Working Group ^[23]^. | 10 |
|  |  | Fees / expert for protocol development ^[12]^. | €2,400.00 |
|  |  | Total (€) | €24,000.00 |
| PROPOSAL 12 TOTAL INVESTMENT | | | €36,936.00 |

Table 17. Proposal 13 Investment Breakdown: Training for parents on the management of haemophilia, in health centres and patient groups.

| Interest groups | Investment description | Investment breakdown | |
| --- | --- | --- | --- |
| National Health System | Economic resources | Informational poster design cost ^[12]^. | €2,000.00 |
|  |  | Number of HTC ^[10]^. | 64 |
|  |  | Number of informational posters ^[6]^. | 2 |
|  |  | Printing cost for each informational poster ^[12]^. | €10.00 |
|  |  | Cost of postage of a package ^[12]^. | €8.00 |
|  |  | Cost of scientific endorsement from the Spanish Society of Haematology and Hemotherapy ^[14]^. | €1,500.00 |
|  |  | Cost of sponsorship from the Spanish Society of Thrombosis and Haemostasis to scientific activities or publications developed by the Pharmaceutical Industry ^[15]^. | €907.50 |
|  |  | Total (€) | €6,199.50 |
| Hospital nursing professionals | Working time spent on training outside of working hours | Number of HTC ^[10]^. | 64 |
|  |  | Number of training sessions per year on the management of HA, for parents of children with HA ^[24]^. | 2 |
|  |  | Duration (hours) of each training session on HA management for parents of children with HA ^[24]^. | 2.50 |
|  |  | Cost per hour of face-to-face training ^[17]^. | €125.00 |
|  |  | Total (€) | €40,000.00 |
| Associations of patients with HA | Human resources | Number of associations of patients with HA in Spain^[25]^. | 20 |
|  |  | Number of annual meetings in each association of patients with HA ^[6]^. | 4 |
|  |  | Duration (hours) of the meetings in the associations of patients with HA ^[6]^. | 2 |
|  |  | Cost per hour of face-to-face training ^[17]^. | €125.00 |
|  |  | Total (€) | €20,000.00 |
| PROPOSAL 13 TOTAL INVESTMENT | | | €66,199.50 |

Table 18. Proposal 14 Investment Breakdown: Training for education professionals about the care of students with haemophilia in schools.

| Interest groups | Investment description | Investment breakdown | |
| --- | --- | --- | --- |
| National Health System | Economic resources | Cost of preparing a guide for education professionals on the management of students with HA ^[12]^. | €4,000.00 |
|  |  | Number of paediatric HA patients (<14 years) ^[1]^. | 479 |
|  |  | Percentage of the paediatric male population (<14 years old) who are 3 to 13 years old ^[26]^. | 81.76% |
|  |  | Number of guidelines on the management of students with HA, by educational centre ^[6]^. | 5 |
|  |  | Printing cost per teacher's guide ^[12]^. | €10.00 |
|  |  | Cost of package postage ^[12]^. | €8.00 |
|  |  | Total (€) | €26,732.07 |
| Hospital nursing professionals | Working time spent on training outside working hours | Number of workshops per year aimed at education professionals ^[6]^. | 19 |
|  |  | Number of hours per workshop for education professionals on the management of students with HA ^[6]^. | 3 |
|  |  | Cost per hour of face-to-face training ^[17]^. | €125.00 |
|  |  | Average cost of room rental for a training session ^[12]^. | €500.00 |
|  |  | Total (€) | €16,625.00 |
| PROPOSAL 14 TOTAL INVESTMENT | | | €43,357.07 |

Table 19. Proposal 15 Investment Breakdown: Training for paediatric patients on adherence to treatment.

| Interest groups | Investment description | Investment breakdown | |
| --- | --- | --- | --- |
| National Health System | Economic resources | Cost of an online training course on therapeutic adherence aimed at patients aged 10 to 13 years old ^[12]^. | €12,952.00 |
|  |  | Total (€) | €12,952.00 |
| Hospital nursing professionals | Working time used to give training | Number of paediatric HA patients (<14 years)^[1]^. | 479 |
|  |  | Percentage of paediatric patients with mild HA ^[1]^. | 44.92% |
|  |  | Percentage of mild paediatric patients receiving prophylaxis treatment ^[19]^. | 4.83% |
|  |  | Percentage of paediatric patients with moderate HA ^[1]^. | 13.56% |
|  |  | Percentage of paediatric patients with moderate HA receiving prophylactic treatment ^[19]^. | 43.33% |
|  |  | Percentage of paediatric patients with severe HA ^[1]^. | 41.53% |
|  |  | Percentage of paediatric patients with severe HA receiving prophylaxis treatment ^[19]^. | 71.50% |
|  |  | Percentage of the paediatric male population (<14 years old) who are 5 to 13 years old ^[26]^. | 68.15% |
|  |  | Number of face-to-face training sessions per year for paediatric HA patients aged 5 to 13 years old^[6]^. | 2 |
|  |  | Number of visits to the nurse equivalent to time spent in each face-to-face training session for paediatric HA patients ^[6,27]^. | 9 |
|  |  | Cost of hospital nursing visits^[4]^. | €42.00 |
|  |  | Total (€) | €93,196.66 |
| Associations of patients with HA | Human resources | Number of associations of patients with HA in Spain ^[25]^. | 20 |
|  |  | Number of annual meetings directed to paediatric patients in each association of patients with HA ^[6]^. | 2 |
|  |  | Duration (hours) of the meetings in the associations of patients with HA ^[6]^. | 2 |
|  |  | Cost per hour of face-to-face training ^[17]^. | € 125.00 |
|  |  | Total (€) | €10,000.00 |
| PROPOSAL 15 TOTAL INVESTMENT | | | €116,148.66 |

## Return

The following tables detail each proposal’s returns and their monetization. The total impact of each return is calculated as follows:

= (return indicator [total amount per proposal]) * (proxy [return value]) * (100% - % deadweight [what would have happened anyway]) * (100% - % return displacement) * (100% - % return attribution [how much of the return was caused by other factors]).

Table 20. Proposal 1 Return Breakdown: Attention by multidisciplinary teams with all specialities involved.

| Return | Return breakdown | | |
| --- | --- | --- | --- |
| Return 1.1. The satisfaction of adult HA patients concerning the NHS would improve. | Indicator | Number of adult HA patients who would improve their satisfaction with the NHS, thanks to access to the nursing service ^[1,28]^. | 1,841 |
|  | Proxy | Average annual premium per private insurance policyholder ^[29]^. | €797.86 |
|  | Deadweight | Percentage of adult patients with haemophilia seen annually by nursing staff ^[2]^. | 46.00% |
|  | Attribution | Percentage of the impact ascribed to returns 1.1, 2.4, 4.3 and 5.5. | 40.00% |
|  |  | Total (€) | €475,804.89 |
| Return 1.2. The empowerment and quality of life of patients with HA would improve, thanks to access to social work services. | Indicator | Number of patients with HA who would be more empowered, thanks to access to social work services ^[1,6]^. | 1,298 |
|  | Proxy | Cost of five family therapy sessions ^[4]^. | €441.51 |
|  | Deadweight | Percentage of haemophilia patients seen annually by social work ^[2]^. | 10.74% |
|  | Attribution | N/A. | 0.00% |
|  |  | Total (€) | €511,342.61 |
| Return 1.3. Oral bleeding would be avoided in patients with HA, thanks to access to dental services. | Indicator | Number of patients with HA in whom the appearance of dental caries would be avoided ^[1,6]^. | 1,298 |
|  | Proxy | Total cost per person associated with the burden of dental disease ^[30]^. | €171.96 |
|  | Deadweight | Percentage of patients with HA who are already being cared for by a dentist ^[6]^. | 50.00% |
|  | Attribution | N/A. | 0.00% |
|  |  | Total (€) | €111,559.05 |
| Return 1.4. The function and quality of life of patients with HA and arthropathy would be improved, thanks to access to rehabilitation/physiotherapy and orthopaedic surgery services. | Indicator | Total number of Quality-Adjusted Life Years (QALY) that would be maintained when attended by a rehabilitation/physiotherapy or orthopaedic surgery professional ^[1,6,31]^. | 52 |
|  | Proxy | Incremental cost-effectiveness threshold per QALY ^[32]^. | €21,000.00 |
|  | Deadweight | Percentage of patients with HA and arthropathy who are already being cared for by an orthopaedic surgery professional ^[6]^. | 50.00% |
|  | Attribution | N/A. | 0.00% |
|  |  | Total (€) | €544,950.00 |
| Return 1.5 The function and quality of life of patients with HA and obesity or overweight would be improved, thanks to access to diet and nutrition services | Indicator | Total number of QALYs that would be gained from being cared for by a dietetic and nutrition professional ^[1,6,8,33]^. | 34 |
|  | Proxy | Incremental cost-effectiveness threshold per QALY ^[32]^. | €21,000.00 |
|  | Deadweight | Percentage of HA patients with obesity or overweight who are already being cared for by a professional in dietetics and nutrition ^[6]^. | 50.00% |
|  | Attribution | N/A. | 0.00% |
|  |  | Total (€) | €355,600.35 |
| Return 1.6 The quality of life of patients with HA and mental health disorders would be improved, thanks to access to psychology services | Indicator | Total number of QALYs that would be gained by being cared for by a professional in psychology ^[1,6,7,33]^. | 92 |
|  | Proxy | Incremental cost-effectiveness threshold per QALY ^[32]^. | €21,000.00 |
|  | Deadweight | Percentage of patients with haemophilia seen annually by psychology ^[2]^. | 15.73% |
|  | Attribution | N/A. | 0.00% |
|  |  | Total (€) | €1,627,800.10 |
| Return 1.7. The satisfaction of informal caregivers of patients with HA concerning the NHS would improve. | Indicator | Number of informal caregivers of patients with HA who would improve their satisfaction with the NHS ^[1,6,28]^. | 969 |
|  | Proxy | Average annual premium per private insurance policyholder ^[29]^. | €797.86 |
|  | Deadweight | Percentage of patients with haemophilia seen annually by nursing staff ^[2]^. | 51.73% |
|  | Attribution | Percentage of the impact ascribed to returns 1.7, 2.6, 4.4 and 5.7. | 40.00% |
|  |  | Total (€) | €223,984.99 |
| PROPOSAL 1 TOTAL RETURN | | | €3,851,041.99 |

Table 21. Proposal 2 Return Breakdown: hospital case manager nurse.

| Return | Return breakdown | | |
| --- | --- | --- | --- |
| Return 2.1. The number of missed specialist appointments of patients with HA would be reduced, thanks to phone calls from the hospital case manager nurse. | Indicator | Total number of missed appointments that could be avoided thanks to phone calls ^[1,3,34]^. | 261 |
|  | Proxy | Cost of follow-up appointment to specialised care. ^[4]^. | €88.30 |
|  | Deadweight | Percentage of nurses with extensive experience in haemophilia (used as a proxy for the level of implantation of a nurse case manager)^[11]^. | 44.00% |
|  | Attribution | N/A. | 0.00% |
|  |  | Total (€) | €12,884.84 |
| Return 2.2. Losses of labour productivity of patients with HA would be reduced, thanks to single-act visits. | Indicator | Total number of hours of labour productivity lost that would be avoided thanks to single-act visits ^[1,3,6]^. | 3,482 |
|  | Proxy | Average profit per normal working hour ^[35]^. | €15.30 |
|  | Deadweight | Percentage of nurses with extensive experience in haemophilia (used as a proxy for the level of implantation of the nurse case manager)^[11]^. | 44.00% |
|  | Attribution | N/A. | 0.00% |
|  |  | Total (€) | €29,827.26 |
| Return 2.3. Out-of-pocket expenses for unnecessary transfers of HA patients would be reduced, thanks to single act consultations. | Indicator | Total number of transfers that would be saved thanks to single-act visits ^[1,3,6]^. | 14,890 |
|  | Proxy | Average cost per transfer to hospital ^[20]^. | €23.91 |
|  | Deadweight | Percentage of nurses with extensive experience in haemophilia (used as a proxy for the level of implantation of the nurse case manager)^[11]^. | 44.00% |
|  | Attribution | N/A. | 0.00% |
|  |  | Total (€) | €199,383.61 |
|  |  |  |  |
| Return 2.4. Satisfaction with the NHS of adult patients with HA would improve. | Indicator | Number of adult patients with HA that would be more satisfied with the NHS thanks to the hospital case manager nurse^[1,6]^. | 423 |
|  | Proxy | Average annual premium per private insurance policyholder^[29]^. | €797.86 |
|  | Deadweight | Percentage of nurses with extensive experience in haemophilia (used as a proxy for the level of implantation of the nurse case manager)^[11]^. | 44.00% |
|  | Attribution | Percentage of the impact ascribed to returns 1.1, 2.4, 4.3 and 5.5. | 70.00% |
|  |  | Total (€) | €56,715.78 |
| Return 2.5. The burden of informal caregivers of patients with HA would be reduced, thanks to single-act visits. | Indicator | Total number of hours of informal care that would be avoided ^[1,3,6]^. | 17,879 |
|  | Proxy | Cost per hour of informal care ^[36]^. | €7.04 |
|  | Deadweight | Percentage of nurses with extensive experience in haemophilia (used as a proxy for the level of implantation of the nurse case manager)^[11]^. | 44.00% |
|  | Attribution | N/A. | 0.00% |
|  |  | Total (€) | €70,484.53 |
| Return 2.6. Informal caregivers of patients with HA would be more satisfied with the NHS. | Indicator | Number of informal caregivers of patients with HA that would be more satisfied with the NHS thanks to the hospital case manager nurse^[1,6]^. | 223 |
|  | Proxy | Average annual premium per private insurance policyholder^[29]^. | €797.86 |
|  | Deadweight | Percentage of nurses with extensive experience in haemophilia (used as a proxy for the level of implantation of the nurse case manager)^[11]^. | 44.00% |
|  | Attribution | Percentage of the impact ascribed to returns 1.7, 2.6, 4.4 and 5.7. | 70.00% |
|  |  | Total (€) | €29,866.32 |
| PROPOSAL 2 TOTAL RETURN | | | €399,162.35 |

Table 22. Proposal 3 Return Breakdown: Training for Primary Care Medicine Professionals on the Management of Comorbidities Related to Aging.

| Return | Return breakdown | | |
| --- | --- | --- | --- |
| Return 3.1. Medical visits to the haematology professional would be reduced, thanks to the fact that PC professionals could manage the comorbidities related to ageing in patients with HA over 65 years of age. | Indicator | Total number of visits related to comorbidities of HA patients older than 65 years that PC medicine professionals could see, instead of haematology professionals ^[1,6,37,38]^. | 1,140 |
|  | Proxy | Difference in cost between follow-up visit to specialist and follow-up visit to PC ^[4]^. | €38.24 |
|  | Deadweight | N/A. | 0.00% |
|  | Attribution | N/A. | 0.00% |
|  |  | Total (€) | €43,605.65 |
| Return 3.2. Out-of-pocket costs for HA patients over 65 years of age, related to travel to follow-up visits for comorbidities related to ageing, would be reduced. | Indicator | Number of HA patients over 65 years old who would reduce their out-of-pocket expenses ^[1,6,37]^. | 52 |
|  | Proxy | Cost of non-health transport per year per patient ^[39]^. | €60.74 |
|  | Deadweight | N/A. | 0.00% |
|  | Attribution | N/A. | 0.00% |
|  |  | Total (€) | €3,152.41 |
| Return 3.3. Self-care and day-to-day activities of patients with HA over 65 would improve | Indicator | Number of HA patients over 65 who would improve their self-care and daily activities, by going to Primary Care instead of Specialised Care to monitor their comorbidities^[1,6,37]^. | 52 |
|  | Proxy | Average annual cost of shopping home delivery services^[12]^. | €96.00 |
|  | Deadweight | N/A. | 0.00% |
|  | Attribution | N/A. | 0.00% |
|  |  | Total (€) | €4,982.40 |
| Return 3.4. The travel time of patients with HA over 65 to attend follow-up visits for their comorbidities would be reduced since visits would be to primary care instead of to the specialist. | Indicator | Number of HA patients over 65 who would reduce travel time to comorbidities follow-up appointments by attending Primary Care instead of Specialised Care. ^[1,6,37]^. | 52 |
|  | Proxy | Average expenditure per person and year on leisure and culture ^[40]^. | €665.73 |
|  | Deadweight | N/A. | 0.00% |
|  | Attribution | N/A. | 0.00% |
|  |  | Total (€) | €34,551.48 |
| Return 3.5. The burden on informal caregivers of HA patients older than 65 would be reduced, thanks to less time used to go with them to follow-up visits for their comorbidities. | Indicator | Number of hours of care that would be reduced thanks to the possibility of having follow-up visits for comorbidities with HA patients over 65 in PC^[1,6,37,38]^. | 513 |
|  | Proxy | Cost per hour of informal care ^[36]^. | €7.04 |
|  | Deadweight | N/A. | 0.00% |
|  | Attribution | N/A. | 0.00% |
|  |  | Total (€) | €3,612.29 |
| PROPOSAL 3 TOTAL RETURN | | | €89,904.22 |

Table 23. Proposal 4 Return Breakdown: Coordination between primary care health centres and haemophilia units through access to medical records throughout the national territory

| Return | Return breakdown | | |
| --- | --- | --- | --- |
| Return 4.1. The mobility of patients with HA would be improved, as they could travel across the country without worrying about their medical history. | Indicator | Number of patients with HA who could travel without worrying about their medical history information. ^[1]^. | 2,595 |
|  | Proxy | Average spending per person and year on trips and overnight stays ^[41]^. | €247.92 |
|  | Deadweight | NHSEHR population coverage ^[42]^. | 93.57% |
|  | Attribution | N/A. | 0.00% |
|  |  | Total (€) | €41,367.56 |
| Return 4.2. The pain of HA patients would be reduced, thanks to better coordination between levels of care. | Indicator | Number of patients with HA who would see their pain reduced. ^[1,43,44]^. | 1,316 |
|  | Proxy | Average expenditure per person and year on leisure and culture ^[40]^. | €665.73 |
|  | Deadweight | NHSEHR population coverage ^[42]^. | 93.57% |
|  | Attribution | Percentage of the impact ascribed to returns 4.2 and 12.3. | 4.77% |
|  |  | Total (€) | €53,664.46 |
| Return 4.3. The satisfaction of adult HA patients with the NHS would improve. | Indicator | Number of adult HA patients who would be more satisfied with the NHS ^[1,45]^. | 1,667 |
|  | Proxy | Average annual premium per private insurance policyholder^[29]^. | €797.86 |
|  | Deadweight | NHSEHR population coverage ^[42]^. | 93.57% |
|  | Attribution | Percentage of the impact ascribed to returns 1.1, 2.4, 4.3 and 5.5. | 95.00% |
|  |  | Total (€) | €4,276.34 |
| Return 4.4. The satisfaction of informal caregivers of patients with HA with the NHS would improve. | Indicator | Number of informal caregivers of patients with HA who would improve their satisfaction with the NHS ^[1,6,45]^. | 878 |
|  | Proxy | Average annual premium per private insurance policyholder^[29]^. | €797.86 |
|  | Deadweight | NHSEHR population coverage ^[42]^. | 93.57% |
|  | Attribution | Percentage of the impact ascribed to returns 1.7, 2.6, 4.4 and 5.7. | 95,00% |
|  |  | Total (€) | €2,251.90 |
| PROPOSAL 4 TOTAL RETURN | | | €101,560.26 |

Table 24. Proposal 5 Return Breakdown: Networking between small and reference centres, and between reference centres with each other.

| Return | Return breakdown | | |
| --- | --- | --- | --- |
| Return 5.1. The referrals of adult patients with mild-moderate HA to other ACs would be reduced. | Indicator | Total number of referrals of adult patients with mild-moderate HA that would be avoided thanks to networking ^[1,6]^. | 660 |
|  | Proxy | Average savings in healthcare resources per mild-moderate adult patient thanks to prior telematic appointment^[6]^. | €9.58 |
|  | Deadweight | Percentage of HA patients living in an Autonomous Community with Reference Centres, Services and Units^[1]^. | 59.00% |
|  | Attribution | N/A. | 0.00% |
|  |  | Total (€) | €2,594.04 |
| Return 5.2. Referrals of adult patients with severe HA and paediatric patients to other ACs would be reduced. | Indicator | Total number of referrals of paediatric and severe HA patients that would be avoided thanks to networking ^[1,6]^. | 902 |
|  | Proxy | Average savings in healthcare resources per seriously ill adult and paediatric patient thanks to prior telematic appointment^[6]^. | €12.82 |
|  | Deadweight | Percentage of HA patients living in an Autonomous Community with Reference Centres, Services and Units^[1]^. | 59.00% |
|  | Attribution | N/A. | 0.00% |
|  |  | Total (€) | €4,740.58 |
| Return 5.3. Referrals of patients with HA to other ACs to be seen by other specialists would be avoided. | Indicator | Number con HA patients referrals that would be avoided thanks to the HTC network. ^[1,6]^. | 1,562 |
|  | Proxy | Average cost of travel to another AC.^[6,46]^. | €66.50 |
|  | Deadweight | Percentage of HA patients living in an Autonomous Community with Reference Centres, Services and Units^[1]^. | 59.00% |
|  | Attribution | N/A. | 0.00% |
|  |  | Total (€) | €42,596.99 |
| Return 5.4. Losses of labour productivity of patients with HA who work would be reduced. | Indicator | Total number of hours of lost labour productivity that would be avoided ^[1,6,43]^. | 891 |
|  | Proxy | Average profit per normal working hour ^[35]^. | €15.30 |
|  | Deadweight | Percentage of HA patients living in an Autonomous Community with Reference Centres, Services and Units^[1]^. | 59.00% |
|  | Attribution | N/A. | 0.00% |
|  |  | Total (€) | €5,588.99 |
| Return 5.5. Adult patients with HA would be more satisfied with the NHS. | Indicator | Number of adult patients with HA who would be more satisfied with the NHS thanks to the HTC network. | 423 |
|  | Proxy | Average annual premium per private insurance policyholder^[29]^. | €797.86 |
|  | Deadweight | Percentage of HA patients living in an Autonomous Community with Reference Centres, Services and Units^[1]^. | 59.00% |
|  | Attribution | Percentage of the impact ascribed to returns 1.1, 2.4, 4.3 and 5.5. | 95.00% |
|  |  | Total (€) | €6,920.68 |
| Return 5.6. The burden of informal caregivers of patients with HA would be reduced. | Indicator | Total number of hours of informal care that would be avoided. | 4,423 |
|  | Proxy | Cost per hour of informal care ^[36]^. | €7.04 |
|  | Deadweight | Percentage of HA patients living in an Autonomous Community with Reference Centres, Services and Units^[1]^. | 59.00% |
|  | Attribution | N/A. | 0.00% |
|  |  | Total (€) | €12,766.39 |
| Return 5.7. Informal caregivers of patients with HA would be more satisfied with the NHS. | Indicator | Number of informal caregivers of patients with HA that would be more satisfied with the NHS thanks to the HTC network. ^[1,6]^. | 223 |
|  | Proxy | Average annual premium per private insurance policyholder^[29]^. | €797.86 |
|  | Deadweight | Percentage of HA patients living in an Autonomous Community with Reference Centres, Services and Units^[1]^. | 59.00% |
|  | Attribution | Percentage of the impact ascribed to returns 1.7, 2.6, 4.4 and 5.7. | 95.00% |
|  |  | Total (€) | €3,644.40 |
| PROPOSAL 5 TOTAL RETURN | | | €78,852.06 |

Table 25. Proposal 6 Return Breakdown: Bringing hospital medication closer to patients.

| Return | Return breakdown | | |
| --- | --- | --- | --- |
| Return 6.1. The out-of-pocket costs associated with the periodic travel to the hospital of patients with moderate or severe HA on prophylaxis, for the collection of medication at the hospital pharmacy, would be reduced. | Indicator | Total number of trips to collect medication that would be avoided ^[1,19,20]^. | 4,263 |
|  | Proxy | Average expenditure on travel to collect medication per patient and year ^[20]^. | €272.60 |
|  | Deadweight | N/A. | 0.00% |
|  | Attribution | N/A. | 0.00% |
|  |  | Total (€) | €1,162,198.68 |
| Return 6.2. The comfort of patients with moderate or severe HA in prophylaxis would be increased. | Indicator | Total number of trips to collect medication that would be avoided ^[1,19,20]^. | 4,263 |
|  | Proxy | Average price of shipping costs ^[12]^. | €10.00 |
|  | Deadweight | N/A. | 0.00% |
|  | Attribution | N/A. | 0.00% |
|  |  | Total (€) | €42,633.85 |
| Return 6.3. Losses of labour productivity in adult patients with moderate or severe HA in prophylaxis, associated with the collection of medication in the hospital pharmacy, would be reduced. | Indicator | Total number of hours of lost labour productivity that would be avoided ^[1,20,43,47]^. | 37 |
|  | Proxy | Average profit per normal working hour ^[35]^. | €15.30 |
|  | Deadweight | N/A. | 0.00% |
|  | Attribution | N/A. | 0.00% |
|  |  | Total (€) | €560.91 |
| Return 6.4. The burden of informal caregivers of patients with moderate or severe HA in prophylaxis, associated with the collection of medication in the hospital pharmacy, would be reduced. | Indicator | Total number of hours of informal care that would be avoided. ^[1,20,43,47]^. | 269 |
|  | Proxy | Cost per hour of informal care ^[36]^. | €7.04 |
|  | Deadweight | N/A. | 0.00% |
|  | Attribution | N/A. | 0.00% |
|  |  | Total (€) | €1,891.98 |
| PROPOSAL 6 TOTAL RETURN | | | €1,207,285.41 |

Table 26. Proposal 7 Return Breakdown: Protocol for the treatment of pain and training for professionals related to the pathology.

| Return | Return breakdown | | |
| --- | --- | --- | --- |
| Return 7.1. The quality of life of patients with mild-moderate HA would be improved. | Indicator | Total number of QALYs that would be gained thanks to pain reduction, in a patient with mild-moderate HA ^[1,48]^. | 241 |
|  | Proxy | Incremental cost-effectiveness threshold per QALY ^[32]^. | €21,000.00 |
|  | Deadweight | Percentage of HA patients who have no pain ^[1,6,44]^. | 50.73% |
|  | Attribution | N/A. | 0.00% |
|  |  | Total (€) | €2,494,404.01 |
| Return 7.2. The quality of life of patients with severe HA would be improved. | Indicator | Total number of QALYs that would be gained thanks to pain reduction, in a patient with severe HA ^[1,48]^. | 63 |
|  | Proxy | Incremental cost-effectiveness threshold per QALY ^[32]^. | €21,000.00 |
|  | Deadweight | Percentage of HA patients who have no pain ^[1,6,44]^. | 50.73% |
|  | Attribution | N/A. | 0.00% |
|  |  | Total (€) | €649,265.57 |
| Return 7.3. The burden on informal caregivers of patients with HA would be reduced, thanks to better pain management. | Indicator | Total number of hours of informal care that would be reduced, thanks to better pain management in patients with HA ^[1,6]^. | 26,738 |
|  | Proxy | Cost per hour of informal care ^[36]^. | €7.04 |
|  | Deadweight | Percentage of HA patients who have no pain^[1,6,44]^. | 50.73% |
|  | Attribution | Percentage of the impact ascribed to returns 7.3 and 12.6. | 0.92% |
|  |  | Total (€) | €91,889.08 |
| PROPOSAL 7 TOTAL RETURN | | | €3,235,558.66 |

Table 27. Proposal 8 Return Breakdown: Early Diagnosis of Arthropathy to Adapt Prophylaxis.

| Return | Return breakdown | | |
| --- | --- | --- | --- |
| Return 8.1. The healthcare costs associated with the management of haemophilic arthropathy in patients with severe HA would be reduced. | Indicator | Number of patients with severe HA in whom signs of subclinical arthropathy would be detected in at least one joint and in whom the development of haemophilic arthropathy would be avoided. ^[1,5,6]^. | 58 |
|  | Proxy | Additional cost of managing an adult with severe haemophilia and at least one target joint (with chronic synovitis)^[49]^. | €791.34 |
|  | Deadweight | N/A. | 0.00% |
|  | Attribution | N/A. | 0.00% |
|  |  | Total (€) | €46,202.44 |
| Return 8.2. The costs associated with the initiation or adjustment of prophylactic treatment of patients with HA would increase. | Indicator | Number of patients with HA in whom subclinical arthropathy would be detected ^[1,5]^. | 357 |
|  | Proxy | Cost of a follow-up visit to a specialist ^[4]^. | -€88.30 |
|  | Deadweight | N/A. | 0.00% |
|  | Attribution | N/A. | 0.00% |
|  |  | Total (€) | -€31,553.23 |
| Return 8.3. The functional status and quality of life of patients with severe HA would be maintained by avoiding the development of haemophilic arthropathy. | Indicator | Total number of QALYs that would be maintained by avoiding the development of haemophilic arthropathy in patients with severe HA ^[1,5,6,31]^. | 16 |
|  | Proxy | Incremental cost-effectiveness threshold per QALY ^[32]^. | €21,000.00 |
|  | Deadweight | N/A. | 0.00% |
|  | Attribution | N/A. | 0.00% |
|  |  | Total (€) | €343,302.62 |
| Return 8.4. Loss of labour productivity in patients with HA, as a consequence of travel to ultrasound follow-up. | Indicator | Number of hours of labour productivity that patients with HA workers would lose associated with visits to radiology for ultrasound follow-up ^[1,3,6,43]^. | 871 |
|  | Proxy | Average profit per normal working hour ^[35]^. | -€15.30 |
|  | Deadweight | N/A. | 0.00% |
|  | Attribution | N/A. | 0.00% |
|  |  | Total (€) | -€13,315.74 |
| Return 8.5. The burden of informal caregivers of patients with HA would increase, as a consequence of going with the patient to radiology consultations for ultrasound follow-up. | Indicator | Number of hours of informal care needed to accompany HA patients to radiology visits for ultrasound follow-up ^[1,3,6]^. | 4,470 |
|  | Proxy | Cost per hour of informal care ^[36]^. | -€7.04 |
|  | Deadweight | N/A. | 0.00% |
|  | Attribution | N/A. | 0.00% |
|  |  | Total (€) | -€31,466.31 |
| PROPOSAL 8 TOTAL RETURN | | | €313,169.78 |

Table 28. Proposal 9 Return Breakdown: Rapid referral protocol between autonomous communities to perform orthopaedic surgeries in reference centres

| Return | Return breakdown | | |
| --- | --- | --- | --- |
| Return 9.1. Healthcare costs would be reduced thanks to a lower number of complications derived from orthopaedic surgery in patients with moderate-severe HA. | Indicator | Total number of complications associated with orthopaedic surgery avoided in patients with HA who underwent surgery in Reference Centres, Services and Units outside the Autonomous Communities in which they live ^[1,43,50,51]^. | 0.94 |
|  | Proxy | Average cost per complication of orthopaedic device or procedure ^[52]^. | €4,128.91 |
|  | Deadweight | Percentage of HA patients living in an Autonomous Community with Reference Centres, Services and Units^[1]^. | 59.00% |
|  | Attribution | N/A. | 0.00% |
|  |  | Total (€) | €1,593.45 |
| Return 9.2. Healthcare costs would be reduced thanks to fewer days of hospitalization for orthopaedic surgery in patients with moderate-severe HA. | Indicator | Total number of days of hospitalization associated with orthopaedic surgery avoided in patients with HA who underwent surgery in Reference Centres, Services and Units outside the Autonomous Communities in which they live^[1,6,43]^. | 37 |
|  | Proxy | Average cost per day of hospitalization for knee replacement ^[53,54]^. | €1,324.07 |
|  | Deadweight | Percentage of HA patients living in an Autonomous Community with Reference Centres, Services and Units^[1]^. | 59.00% |
|  | Attribution | N/A. | 0.00% |
|  |  | Total (€) | €20,267.08 |
| Return 9.3. The functional status and quality of life of patients with HA and indication for orthopaedic surgery would be improved, thanks to an optimal result of the surgery. | Indicator | Total number of QALYs that would be maintained thanks to an optimal result of orthopaedic surgery ^[1,31,43,50,51]^. | 0.11 |
|  | Proxy | Incremental cost-effectiveness threshold per QALY ^[32]^. | €21,000.00 |
|  | Deadweight | Percentage of HA patients living in an Autonomous Community with Reference Centres, Services and Units^[1]^. | 59.00% |
|  | Attribution | N/A. | 0.00% |
|  |  | Total (€) | €972.53 |
| Return 9.4. The out-of-pocket costs associated with travelling to other Autonomous Communities with the HA patient would increase. | Indicator | Total number of kilometres travelled by a caregiver to accompany the patient with HA who underwent orthopaedic surgery in Reference Centres, Services and Units outside their Autonomous Communities of residence^[1,6,43]^. | 3,823 |
|  | Proxy | Cost per kilometre travelled ^[46]^. | -€0.19 |
|  | Deadweight | Percentage of HA patients living in an Autonomous Community with Reference Centres, Services and Units ^[1]^. | 59.00% |
|  | Attribution | N/A. | 0.00% |
|  |  | Total (€) | -€297.78 |
| Return 9.5. The out-of-pocket expenses associated with spending the night with the HA patient in an Autonomous Communities other than their residence would increase. | Indicator | Total number of days in which the patient with HA who underwent orthopaedic surgery will be accompanied in Reference Centres, Services and Units outside their Autonomous Communities of residence ^[1,6,43,54]^. | 64 |
|  | Proxy | Average daily spending per person in overnight stay ^[55]^. | -€112.29 |
|  | Deadweight | Percentage of HA patients living in an Autonomous Community with Reference Centres, Services and Units ^[1]^. | 59.00% |
|  | Attribution | N/A. | 0.00% |
|  |  | Total (€) | -€2,95.,59 |
| PROPOSAL 9 TOTAL RETURN | | | €19,583.68 |

Table 29. Proposal 11 Return Breakdown: Training for patients with inhibitor, and their families, on the management and care of haemophilia

| Return | Return breakdown | | |
| --- | --- | --- | --- |
| Return 11.1 Medical visits to the hospital would be reduced, thanks to better management of the disease. | Indicator | Total number of hospital visits of patients with prevalent HA and inhibitor would be reduced ^[1,6,43]^. | 192 |
|  | Proxy | Cost of a follow-up appointment with a specialist ^[4]^. | €88.30 |
|  | Deadweight | Percentage of patients who consider that training would not affect the number of hospital visits. ^[56]^. | 41.00% |
|  | Attribution | N/A. | 0.00% |
|  |  | Total (€) | €9,982.04 |
| Return 11.2. The emotional state of patients with HA and inhibitor would be improved, thanks to better management of complications associated with the inhibitor. | Indicator | Number of patients with HA and inhibitor (incidental and prevalent) that would improve their emotional state ^[1,43,57–59]^. | 116 |
|  | Proxy | Average annual cost for psychological follow-up of patients with HA and inhibitor^[4,6]^. | €176.60 |
|  | Deadweight | Percentage of inhibitor patients with no anxiety. ^[44]^. | 60.37% |
|  | Attribution | N/A. | 0.00% |
|  |  | Total (€) | €8,153.77 |
| Return 11.3. Trauma would be avoided in patients with prevalent HA and inhibitor, thanks to training in the prevention of those situations and behaviours that involve high risk. | Indicator | Total number of bleeds that would be avoided by preventing trauma in patients with prevalent HA and inhibitor ^[1,43,59,60]^. | 2 |
|  | Proxy | Average cost of treating mild-moderate bleeding ^[61]^. | €8,852.10 |
|  | Deadweight | N/A. | 0.00% |
|  | Attribution | N/A. | 0.00% |
|  |  | Total (€) | €17,152.34 |
| Return 11.4. The mobility of patients with prevalent HA and inhibitor would be improved, thanks to better self-care and adherence after received training. | Indicator | Number of patients with prevalent HA and inhibitor that would improve their mobility ^[1,43,59]^. | 26 |
|  | Proxy | Cost of a wheelchair ^[62]^. | €258.15 |
|  | Deadweight | Percentage of patients with inhibitor who do not have impaired mobility ^[44]^. | 15.66% |
|  | Attribution | N/A. | 0.00% |
|  |  | Total (€) | €5,631.75 |
| Return 11.5. The emotional state (stress) of the informal caregivers of patients with incidental HA and inhibitor would be improved. | Indicator | Number of informal caregivers of patients with incidental HA and inhibitor who would improve their emotional state ^[1,6,43,57–59]^. | 91 |
|  | Proxy | Cost of five sessions of psychological therapy ^[4]^. | €441.51 |
|  | Deadweight | Percentage of informal caregivers of paediatric HA patients who stated that HA had not affected their life ^[63]^. | 33.80% |
|  | Attribution | N/A. | 0.00% |
|  |  | Total (€) | €26,488.57 |
| Return 11.6. The burden of informal caregivers of patients with incidental HA and inhibitor would increase when accompanying them to face-to-face training workshops. | Indicator | Total number of hours of informal care that would be increased for informal caregivers of patients with incidental HA and inhibitor ^[1,6,43,57,58]^. | 1,007 |
|  | Proxy | Cost per hour of informal care ^[36]^. | -€7.04 |
|  | Deadweight | N/A. | 0.00% |
|  | Attribution | N/A. | 0.00% |
|  |  | Total (€) | -€7,089.05 |
| Return 11.7. The burden on informal caregivers of patients with prevalent HA and inhibitor would be reduced, thanks to the reduction in hospital visits. | Indicator | Total number of hours of informal care that would be reduced for informal caregivers of patients with prevalent HA and inhibitor ^[1,6,43,56,59]^. | 23 |
|  | Proxy | Cost per hour of informal care ^[36]^. | €7.04 |
|  | Deadweight | N/A. | 0.00% |
|  | Attribution | N/A. | 0.00% |
|  |  | Total (€) | €161.16 |
| PROPOSAL 11 TOTAL RETURN | | | €60,480.57 |

Table 30. Proposal 12 Return Breakdown: Inhibitor eradication protocols.

| Return | Return breakdown | | |
| --- | --- | --- | --- |
| Return 12.1. The direct healthcare costs related to the treatment of patients with severe HA would be reduced, thanks to greater success in the inhibitor eradication | Indicator | Number of patients with severe HA and incidental inhibitor in whom the inhibitor could be eradicated with ITI treatment, after the implementation of the eradication protocol ^[1,6,57,58]^. | 198 |
|  | Proxy | Difference in mean cost of treating a patient with an inhibitor versus one without an inhibitor ^[61,64]^. | €393,590.36 |
|  | Deadweight | Percentage of patients with already successful ITI treatment^[1,6,43,57,58]^. | 93.59% |
|  | Attribution | N/A. | 0.00% |
|  |  | Total (€) | €5,006,469.33 |
| Return 12.2. The mobility of patients with HA and inhibitor would be improved, thanks to better joint health after the eradication of the inhibitor. | Indicator | Number of patients with HA and inhibitor who would improve their mobility, thanks to the eradication of the inhibitor ^[1,6,43,44,57,58]^. | 134 |
|  | Proxy | Cost of a wheelchair ^[62]^. | €258.15 |
|  | Deadweight | Percentage of patients with already successful ITI treatment^[1,6,43,57,58]^. | 93.59% |
|  | Attribution | N/A. | 0.00% |
|  |  | Total (€) | €2,224.37 |
| Return 12.3. The pain and discomfort of patients with HA and inhibitor would be reduced, thanks to more successful inhibitor eradication. | Indicator | Number of patients with HA and inhibitor who would reduce their pain and discomfort thanks to more successful inhibitor eradication ^[1,6,43,44,57,58]^. | 90 |
|  | Proxy | Average expenditure per person and year on leisure and culture ^[40]^. | €665.73 |
|  | Deadweight | Percentage of patients with already successful ITI treatment^[1,6,43,57,58]^. | 93.59% |
|  | Attribution | Percentage of the impact ascribed to returns 4.2 and 12.3. | 30.00% |
|  |  | Total (€) | €2,688.83 |
| Return 12.4. Improvement of day-to-day activities performance of patients with HA and inhibitor, thanks to inhibitor eradication. | Indicator | Number of patients with HA and inhibitor for whom day-to-day activities performance would improve^[1,6,43,44,57,58]^. | 87 |
|  | Proxy | Average annual cost of shopping home delivery service^[12]^. | €96.00 |
|  | Deadweight | Percentage of patients with already successful ITI treatment^[1,6,43,57,58]^. | 93.59% |
|  | Attribution | N/A. | 0.00% |
|  |  | Total (€) | €538.41 |
| Return 12.5. The time needed by patients with incidental HA and inhibitor to comply with the inhibitor eradication treatment (more visits, more frequent doses) would increase. | Indicator | Number of patients with incidental HA and inhibitor for whom the time to comply with the inhibitor eradication treatment would increase. ^[1,6,43,57,58]^. | 262 |
|  | Proxy | Average spending per person and year on games and hobbies ^[65]^. | -€18.88 |
|  | Deadweight | Percentage of patients with already successful ITI treatment^[1,6,43,57,58]^. | 93.59% |
|  | Attribution | N/A. | 0.00% |
|  |  | Total (€) | -€316.86 |
| Return 12.6. The burden on informal caregivers of patients with HA and inhibitor would be reduced, thanks to the eradication of the inhibitor. | Indicator | Total number of hours of informal care for patients with HA and inhibitor that would be reduced ^[1,6,57,58,66]^. | 24,971 |
|  | Proxy | Cost per hour of informal care ^[36]^. | €7.04 |
|  | Deadweight | Percentage of patients with already successful ITI treatment^[1,6,43,57,58]^. | 93.59% |
|  | Attribution | Percentage of the impact ascribed to returns 7.3 and 12.6. | 30.00% |
|  |  | Total (€) | €7,888.38 |
| Return 12.7. The burden on informal caregivers of patients with incidental HA and inhibitor would increase due to the increase in the time required to comply with the inhibitor eradication treatment (more visits, more frequent doses). | Indicator | Increase of informal care hours for patients with incidental HA and inhibitor. ^[1,6,43,57,58]^. | 95,561 |
|  | Proxy | Cost per hour of informal care ^[36]^. | -€7.04 |
|  | Deadweight | Percentage of patients with already successful ITI treatment^[1,6,43,57,58]^. | 93.59% |
|  | Attribution | N/A. | 0.00% |
|  |  | Total (€) | -€43,125.06 |
| PROPOSAL 12 TOTAL RETURN | | | €4,976,367.41 |

Table 31. Proposal 13 Return Breakdown: Training for parents on the management of haemophilia, in health centres and patient groups.

| Return | Return breakdown | | |
| --- | --- | --- | --- |
| Return 13.1. The proportion of overweight or obese paediatric HA patients would be reduced, thanks to a more active life. | Indicator | Number of paediatric HA patients who would improve their body mass index thanks to a more active life ^[1,67]^. | 364 |
|  | Proxy | Average expenditure per person and year on goods and services related to sport ^[68]^. | €119.40 |
|  | Deadweight | Percentage of paediatric haemophilia patients who are neither obese nor overweight ^[8]^. | 81.20% |
|  | Attribution | N/A. | 0.00% |
|  |  | Total (€) | €8,178.32 |
| Return 13.2. The social relationships of paediatric HA patients would improve, thanks to a life with greater social interaction. | Indicator | Number of paediatric HA patients who would improve their social relationships thanks to a more active life ^[1,67]^. | 364 |
|  | Proxy | Annual spending on extracurricular activities ^[69]^. | €720.00 |
|  | Deadweight | Percentage of paediatric HA patients who say they don’t have problems making friends. ^[70]^. | 67.60% |
|  | Attribution | Percentage of the impact ascribed to returns 13.2, 14.2 and 15.3. | 60.00% |
|  |  | Total (€) | €34,000.86 |
| Return 13.3. The emotional state of informal caregivers of paediatric HA patients would improve, thanks to better management of the pathology. | Indicator | Number of informal caregivers of paediatric HA patients who would improve their emotional well-being thanks to received training^[1,6,67]^. | 364 |
|  | Proxy | Cost of five sessions of psychological therapy ^[4]^. | €441.51 |
|  | Deadweight | Percentage of informal caregivers of paediatric HA patients who stated that HA had not affected their life ^[63]^. | 33.80% |
|  | Attribution | Percentage of the impact ascribed to returns 13.3, 14.4 and 15.5. | 66.67% |
|  |  | Total (€) | €35,496.16 |
| Return 13.4. The family dynamics would improve, thanks to fewer mistakes in the child’s HA management. | Indicator | Number of families with children with HA who would improve their family functioning thanks to received training^[1,67]^. | 407 |
|  | Proxy | Cost of five family therapy sessions ^[4]^. | €441.51 |
|  | Deadweight | Percentage of informal caregivers of paediatric HA patients who stated that HA had not affected their life ^[63]^. | 33.80% |
|  | Attribution | Percentage of the impact ascribed to returns 13.4 and 15.7. | 50.00% |
|  |  | Total (€) | €59,549.48 |
| PROPOSAL 13 TOTAL RETURN | | | €137,224.82 |

Table 32. Proposal 14 Return Breakdown: Training for education professionals about the care of students with haemophilia in schools

| Return | Return breakdown | | |
| --- | --- | --- | --- |
| Return 14.1. School delay in paediatric HA patients due to missed school hours would be avoided, thanks to the training of education professionals in the management of the student with HA. | Indicator | School delay in paediatric HA patients due to missed school hours would be avoided, thanks to the training of education professionals in the management of the student with HA^[1,6,26,71,72]^. | 3,245 |
|  | Proxy | Average cost/hour of private tutoring for school support ^[6]^. | €11.00 |
|  | Deadweight | Percentage of teachers trained in the management of students with HA ^[6]^. | 10.00% |
|  | Attribution | N/A. | 0.00% |
|  |  | Total (€) | €32,127.47 |
| Return 14.2. The social relationships of paediatric HA patients would be improved, thanks to better school integration. | Indicator | Number of paediatric HA patients who would improve their school integration, thanks to education professionals training ^[1,26,70,71]^. | 58 |
|  | Proxy | Average annual spending on children's birthdays by family ^[6]^. | €120.00 |
|  | Deadweight | Percentage of teachers trained in the management of students with HA ^[6]^. | 10.00% |
|  | Attribution | Percentage of the impact ascribed to returns 13.2, 14.2 and 15.3. | 70.00% |
|  |  | Total (€) | €1,892.82 |
| Return 14.3. The physical activity of students with HA would be improved, thanks to more participation in school sports activities. | Indicator | Number of paediatric HA patients who would participate in school physical activities, thanks to educational professionals training^[1,26,71,73]^. | 97 |
|  | Proxy | Average expenditure per person and year on goods and services related to sport ^[68]^. | €119.40 |
|  | Deadweight | Percentage of teachers trained in the management of students with HA ^[6]^. | 10.00% |
|  | Attribution | N/A. | 0.00% |
|  |  | Total (€) | €10,461.87 |
| Return 14.4. The emotional state of the informal caregivers of paediatric HA patients would be improved, thanks to the correct management of their children’s disease in schools. | Indicator | Number of informal caregivers of paediatric HA patients who would improve their emotional well-being, thanks to education professionals training^[1,6,26,63]^. | 259 |
|  | Proxy | Cost of five sessions of psychological therapy ^[4]^. | €441.51 |
|  | Deadweight | Percentage of teachers trained in the management of students with HA ^[6]^. | 10.00% |
|  | Attribution | Percentage of the impact ascribed to returns 13.3, 14.4 and 15.5. | 66.67% |
|  |  | Total (€) | €34,366.31 |
| Return 14.5. The burden on informal caregivers of paediatric HA patients would be reduced. | Indicator | Number of hours of informal care that would be avoided ^[1,6,26]^. | 1,568 |
|  | Proxy | Cost per hour of informal care ^[36]^. | €7.04 |
|  | Deadweight | Percentage of teachers trained in the management of students with HA ^[6]^. | 10.00% |
|  | Attribution | N/A. | 0.00% |
|  |  | Total (€) | €9,933.13 |
| PROPOSAL 14 TOTAL RETURN | | | €88,781.61 |

Table 33. Proposal 15 Return Breakdown: Training for paediatric patients on adherence to treatment.

| Return | Return breakdown | | |
| --- | --- | --- | --- |
| Return 15.1. The physical condition of paediatric HA patients would be improved and pain would be reduced, thanks to increased adherence. | Indicator | Number of QALYs that would be maintained in paediatric HA patients that would prevent pain, thanks to increased adherence ^[1,6,19,26,48]^. | 9 |
|  | Proxy | Incremental cost-effectiveness threshold per QALY ^[32]^. | €21,000.00 |
|  | Deadweight | Percentage of patients with HA 5 to 13 years who are adherent ^[1,26,74]^. | 85.99% |
|  | Attribution | N/A. | 0.00% |
|  |  | Total (€) | €25,029.24 |
| Return 15.2. The mood of paediatric HA patients would improve, thanks to a better physical condition and greater control of the disease. | Indicator | Number of paediatric HA patients who would improve their mood, thanks to increased adherence ^[1,6,19]^. | 62 |
|  | Proxy | Cost of five sessions of psychological therapy ^[4]^. | €441.51 |
|  | Deadweight | Percentage of patients with HA 5 to 13 years who are adherent ^[1,26,74]^. | 85.99% |
|  | Attribution | N/A. | 0.00% |
|  |  | Total (€) | €3,813.22 |
| Return 15.3. The social relationships of paediatric HA patients would be improved, thanks to a better physical condition and greater control of the disease. | Indicator | Number of paediatric HA patients who would improve their social relationships, thanks to increased adherence ^[1,6,19]^. | 62 |
|  | Proxy | Average annual spending on children's birthdays by their families ^[6]^. | €120.00 |
|  | Deadweight | Percentage of patients with HA 5 to 13 years who are adherent ^[1,26,74]^. | 85.99% |
|  | Attribution | Percentage of the impact ascribed to returns 13.2, 14.2 and 15.3. | 70.00% |
|  |  | Total (€) | €310.92 |
|  |  |  |  |
| Return 15.4. The days of school missed by paediatric HA patients would be reduced, thanks to a better physical condition and greater control of the pathology. | Indicator | Number of missed school hours that would be avoided in paediatric HA patients thanks to increased adherence ^[1,6,19,72]^. | 1,109 |
|  | Proxy | Average cost/hour of private tutoring for school support ^[6]^. | €11.00 |
|  | Deadweight | Percentage of patients with HA 5 to 13 years who are adherent ^[1,26,74]^. | 85.99% |
|  | Attribution | N/A. | 0.00% |
|  |  | Total (€) | €1,710.07 |
| Return 15.5. The emotional state of informal caregivers of paediatric HA patients would be improved, thanks to better management of the pathology. | Indicator | Number of informal caregivers of paediatric HA patients who would improve their emotional well-being thanks to increased adherence ^[1,6,19,26]^. | 62 |
|  | Proxy | Cost of five sessions of psychological therapy ^[4]^. | €441.51 |
|  | Deadweight | Percentage of patients with HA 5 to 13 years who are adherent ^[1,26,74]^. | 85.99% |
|  | Attribution | Percentage of the impact ascribed to returns 13.3, 14.4 and 15.5. | 66.67% |
|  |  | Total (€) | €1,271.07 |
| Return 15.6. The burden on informal caregivers of paediatric HA patients would be reduced, thanks to better disease control. | Indicator | Number of hours of informal care that would be reduced for informal caregivers of paediatric HA patients thanks to increased adherence ^[1,6,19,26,72]^. | 2,663 |
|  | Proxy | Cost per hour of informal care ^[36]^. | €7.04 |
|  | Deadweight | Percentage of patients with HA from 5 to 13 years who are adherent ^[1,26,74]^. | 85.99% |
|  | Attribution | N/A. | 0.00% |
|  |  | Total (€) | €2,626.67 |
| Return 15.7. Family dynamics would improve, thanks to better adherence. | Indicator | Number of families with children with HA who would improve their family dynamics thanks to improved adherence ^[1,6,19]^. | 62 |
|  | Proxy | Cost of five family therapy sessions ^[4]^. | €441.51 |
|  | Deadweight | Percentage of patients with HA 5 to 13 years who are adherent ^[1,26,74]^. | 85.99% |
|  | Attribution | Percentage of the impact ascribed to returns 13.4 and 15.7. | 50.00% |
|  |  | Total (€) | €1,906.61 |
| PROPOSAL 15 TOTAL RETURN | | | €36,667.82 |

# List of assumptions

The following tables include the assumptions that were used in the SROI analysis, but were not included in the sensitivity analysis.

Table 34. Assumptions of Proposal 1. Attention from multidisciplinary teams with all specialities involved.

| Assumption | Datum |
| --- | --- |
| Percentage of patients with HA who would reduce their body mass index after an intervention with exercise and diet. | 50% |
| Percentage of patients with HA in whom the development of dental caries would be avoided. | 50% |
| Percentage of patients with HA and arthropathy who are already being cared for by an orthopaedic surgical professional. | 50% |
| Percentage of patients with HA who would improve their mental health status when treated by psychology services. | 50% |
| Percentage of patients who would improve their empowerment thanks to access to social work services. | 50% |

Table 35. Assumptions of Proposal 2. Hospital case manager nurse.

| Assumption | Datum |
| --- | --- |
| Number of telephone calls per year per patient for the follow-up of patients with mild-moderate HA. | 2 |
| Number of telephone calls per year per patient for the follow-up of patients with severe HA and paediatric patients. | 4 |
| Number of visits to Specialist Care Services that HA patients would save thanks to single-act visits. | 4 |
| Time (hours) to attend each specialised care visit. * | 2.5 |

*This assumption is shared with proposals 2, 8 and 11.

Table 36. Assumptions of Proposal 3. Training for primary care medical professionals on the management of ageing-related comorbidities.

| Assumption | Datum |
| --- | --- |
| Number of face-to-face sessions per year per HTC, for the training of primary care medical professionals in the management of ageing-related comorbidities. | 1 |
| Duration (hours) of face-to-face sessions for the training of primary care professionals. | 4 |
| Number of hours per visit that caregivers of HA patients over 65 would save by accompanying them to primary care instead of specialised care. | 1.5 |

Table 37. Assumptions of Proposal 5. Networking between small and reference centres, and between reference centres with each other

| Assumption | Datum |
| --- | --- |
| Number of annual posts on the intranet blog for networking | 4 |
| Probability of being referred to another HTC after telematic consultation in adults with mild and moderate HA. | 10% |
| Probability of being referred to another HTC after telematic consultation in adults with severe HA and paediatric patients. | 20% |
| Average number of consultations per patient and year in another HTC for adult patients with mild-moderate HA. | 0.5 |
| Average number of consultations per patient and year in another HTC for adult patients with severe HA and paediatric patients. | 1.0 |

Table 38. Assumptions of Proposal 7. Protocol for pain treatment and training for professionals related to the pathology

| Assumption | Datum |
| --- | --- |
| Number of sessions by HTC of continuous face-to-face training per year in HA, aimed at healthcare professionals in pain units. | 1 |
| Duration (hours) of training for healthcare professionals in HA pain units (2 days, 4 hours a day). | 8 |
| Number of hours of informal care that would be reduced thanks to better pain control and management of patients with HA. | 24 |

Table 39. Assumptions of Proposal 8. Early diagnosis of arthropathy to adapt prophylaxis

| Assumption | Datum |
| --- | --- |
| Time (hours) to attend each specialised care visit. * | 2.5 |

*This assumption is shared with proposals 2, 8 and 11.

Table 40. Assumptions of Proposal 10. Development of a patient national registry

| Assumption | Datum |
| --- | --- |
| Number of members of the national registry coordination group. | 21 |
| Number of experts needed for the national registry multidisciplinary expert committee. | 5 |
| Total number of talks needed on the importance of the national registry (one for each autonomous community/city). | 19 |

Table 41. Assumptions of Proposal 11 Training for patients with inhibitor, and their families, on the management and care of haemophilia

| Assumption | Datum |
| --- | --- |
| Number of informative posters. | 2 |
| Number of workshops per year by HTC aimed at patients with incidental HA and inhibitor and their families. | 1 |
| Duration (hours) of the workshops aimed at patients with incidental HA and inhibitor and their families. | 3 |
| Mean visits to the psychologist of adult HA patients with inhibitor. | 2 |
| Time (hours) to attend each specialised care visit. * | 2.5 |

*This assumption is shared with proposals 2, 8 and 11.

Table 42. Assumptions of Proposal 13. Training for parents on the management of haemophilia, delivered in health centres and patient groups

| Assumption | Datum |
| --- | --- |
| Number of annual meetings in each association of patients with HA. | 4 |
| Duration (hours) of the meetings in the associations of patients with HA. | 2 |

Table 43. Assumptions of Proposal 14. Training for education professionals about the care of students with haemophilia in schools.

| Assumption | Datum |
| --- | --- |
| Number of workshops per year aimed at education professionals (one for each autonomous community/city). | 19 |
| Number of hours per workshop for education professionals on the management of students with HA. | 3 |
| Number of guidelines for managing the student with HA by the educational centre. | 5 |
| Number of hours invested by caregivers of patients with HA in training teachers on the management of students with HA. | 3 |
| Number of hours spent on travel to/from school. | 1 |
| Average cost/hour of private tutoring for school support. * | €11 |
| Average annual spending on children's birthdays by family. * | €120 |

*This assumption is shared with proposals14 and 15.

Table 44. Assumptions of Proposal 15. Training for paediatric patients on adherence to treatment

| Assumption | Datum |
| --- | --- |
| Number of face-to-face training sessions per year for paediatric HA patients aged 5 to 13 years old. | 2 |
| Number of nursing visits equivalent to the time spent in each face-to-face training session for paediatric HA patients. | 9 |
| Number of annual meetings in each association of patients with HA aimed at paediatric patients. | 2 |
| Average cost/hour of private tutoring for school support. * | €11 |
| Average annual spending on children's birthdays per family. * | €120 |

*This assumption is shared with proposals14 and 15.

The following tables include the assumptions that were used in the SROI analysis and were included in the sensitivity analysis (worst-case scenario, reference case, and best-case scenario).

Table 45. Sensitivity analysis. Assumption 1.

| Assumption included in the calculation (investment) | Reference case | Worst-case scenario | Best-case scenario |
| --- | --- | --- | --- |
| Percentage of patients with HA who are already been seen by a dentist. | 50% | 25% | 75% |
| Assumption included in the calculation (return) | Reference case | Worst-case scenario | Best-case scenario |
| Percentage of patients with HA who are already been seen by a dentist. | 50% | 75% | 25% |
| Proposals and returns that include this assumption | | | |
| Proposal 1. Attention from multidisciplinary teams with all specialities involved.   - Return 1.3. Oral bleeding would be avoided in patients with HA, thanks to access to dental services. | | | |

Table 46. Sensitivity analysis. Assumption 2.

| Assumption included in the calculation (return) | Reference case | Worst-case scenario | Best-case scenario |
| --- | --- | --- | --- |
| Percentage of patients with HA that would improve their auricular health status when treated by rehabilitation or an orthopaedic surgical professional. | 50% | 25% | 75% |
| Proposals and returns that include this assumption | | | |
| Proposal 1. Attention from multidisciplinary teams with all specialities involved.   - Return 1.4. The function and quality of life of patients with HA and arthropathy would be improved, thanks to access to rehabilitation/physiotherapy and orthopaedic surgery services. | | | |

Table 47. Sensitivity analysis. Assumption 3.

| Assumption included in the calculation ((investment) | Reference case | Worst-case scenario | Best-case scenario |
| --- | --- | --- | --- |
| Percentage of patients with HA and obesity or overweight who are already being cared for by a professional in dietetics and nutrition. | 50% | 25% | 75% |
| Assumption included in the calculation (return) | Reference case | Worst-case scenario | Best-case scenario |
| Percentage of patients with HA and obesity or overweight who are already being cared for by a professional in dietetics and nutrition. | 50% | 75% | 25% |
| Proposals and returns that include this assumption | | | |
| Proposal 1. Attention from multidisciplinary teams with all specialities involved.   - Return 1.5 The function and quality of life of patients with HA and obesity or overweight would be improved, thanks to access to diet and nutrition services | | | |

Table 48. Sensitivity analysis. Assumption 4.

| Assumption included in the calculation (return) | Reference case | Worst-case scenario | Best-case scenario |
| --- | --- | --- | --- |
| Incremental cost-effectiveness threshold per QALY. | €21,000 | €11,000 | €30,000 |
| Proposals and returns that include this assumption | | | |
| Proposal 1. Attention from multidisciplinary teams with all specialities involved.   - Return 1.4. The function and quality of life of patients with HA and arthropathy would be improved, thanks to access to rehabilitation/physiotherapy and orthopaedic surgery services. - Return 1.5 The function and quality of life of patients with HA and obesity or overweight would be improved, thanks to access to diet and nutrition services - Return 1.6 The quality of life of patients with HA and mental health disorders would be improved, thanks to access to psychology services | | | |
| Proposal 7. Protocol for pain treatment and training for professionals related to the pathology   - Return 7.1. The quality of life of patients with mild-moderate HA would be improved. - Return 7.2. The quality of life of patients with severe HA would be improved. | | | |
| Proposal 8. Early diagnosis of arthropathy to adapt prophylaxis   - Return 8.3. The functional status and quality of life of patients with severe HA would be maintained by avoiding the development of haemophilic arthropathy. | | | |
| Proposal 9. Rapid referral protocol between autonomous communities to perform orthopaedic surgeries in reference centres   - Return 9.3. The functional status and quality of life of patients with HA and indication for orthopaedic surgery would be improved, thanks to an optimal result of the surgery. | | | |
| Proposal 15. Training for paediatric patients on adherence to treatment.   - Return 15.1. The physical condition of paediatric HA patients would be improved and pain would be reduced, thanks to increased adherence. | | | |

Table 49. Sensitivity analysis. Assumption 5.

| Assumption included in the calculation (return) | Reference case | Worst-case scenario | Best-case scenario |
| --- | --- | --- | --- |
| Percentage of patients with HA with an informal caregiver. | 42.9% | 26.6% | 51.1% |
| Proposals and returns that include this assumption | | | |
| Proposal 1. Attention from multidisciplinary teams with all specialities involved.   - Return 1.7. The satisfaction of informal caregivers of patients with HA concerning the NHS would improve. | | | |
| Proposal 2. Hospital case manager nurse.   - Return 2.6. Informal caregivers of patients with HA would be more satisfied with the NHS. | | | |
| Proposal 4. Coordination between primary care health centres and haemophilia units through access to medical records throughout the national territory.   - Return 4.4. The satisfaction of informal caregivers of patients with HA with the NHS would improve. | | | |
| Proposal 5. Networking between small and reference centres, and between reference centres with each other.   - Return 5.7. Informal caregivers of patients with HA would be more satisfied with the NHS. | | | |
| Proposal 7. Protocol for the treatment of pain and training for professionals related to the pathology.   - Return 7.3. The burden on informal caregivers of patients with HA would be reduced, thanks to better pain management. | | | |
| Proposal 9. Rapid referral protocol between autonomous communities to perform orthopaedic surgeries in reference centres.   - Return 9.4. The out-of-pocket costs associated with travelling to other Autonomous Communities with the HA patient would increase - Return 9.5. The out-of-pocket expenses associated with spending the night with the HA patient in an Autonomous Communities other than their residence would increase. | | | |
| Proposal 12. Inhibitor eradication protocols.   - Return 12.6. The burden on informal caregivers of patients with HA and inhibitor would be reduced, thanks to the eradication of the inhibitor. | | | |

Table 50. Sensitivity analysis. Assumption 6.

| Assumption included in the calculation (return) | Reference case | Worst-case scenario | Best-case scenario |
| --- | --- | --- | --- |
| Number of adult HA patients who would improve their satisfaction with the NHS thanks to the hospital case nurse manager. | 20% | 10% | 30% |
| Proposals and returns that include this assumption | | | |
| Proposal 2. Hospital case manager nurse.   - Return 2.4. Satisfaction with the NHS of adult patients with HA would improve. - Return 2.6. Informal caregivers of patients with HA would be more satisfied with the NHS. | | | |

Table 51. Sensitivity analysis. Assumption 7.

| Assumption included in the calculation (return) | Reference case | Worst-case scenario | Best-case scenario |
| --- | --- | --- | --- |
| Percentage of adult patients with HA with an informal caregiver. | 30% | 10% | 40% |
| Proposals and returns that include this assumption | | | |
| Proposal 2. Hospital case manager nurse.   - Return 2.5. The burden of informal caregivers of patients with HA would be reduced, thanks to single-act visits. | | | |
| Proposal 3. Training for Primary Care Medicine Professionals on the Management of Comorbidities Related to Aging.   - Return 3.5. The burden on informal caregivers of HA patients older than 65 would be reduced, thanks to less time used to go with them to follow-up visits for their comorbidities. | | | |
| Proposal 5. Networking between small and reference centres, and between reference centres with each other.   - Return 5.6. The burden of informal caregivers of patients with HA would be reduced. | | | |
| Proposal 6. Bringing hospital medication closer to patients.   - Return 6.4. The burden of informal caregivers of patients with moderate or severe HA in prophylaxis, associated with the collection of medication in the hospital pharmacy, would be reduced. | | | |
| Proposal 8. Early Diagnosis of Arthropathy to Adapt Prophylaxis.   - Return 8.5. The burden of informal caregivers of patients with HA would increase, as a consequence of going with the patient to radiology consultations for ultrasound follow-up. | | | |
| Proposal 11. Training for patients with inhibitor, and their families, on the management and care of haemophilia   - Return 11.7. The burden on informal caregivers of patients with prevalent HA and inhibitor would be reduced, thanks to the reduction in hospital visits. | | | |

Table 52. Sensitivity analysis. Assumption 8.

| Assumption included in the calculation (return) | Reference case | Worst-case scenario | Best-case scenario |
| --- | --- | --- | --- |
| Percentage of medical visits related to comorbidities of patients with AH over 65 years of age, which could be attended by the primary care professional instead of the haematology professional. | 50% | 25% | 75% |
| Proposals and returns that include this assumption | | | |
| Proposal 3. Training for Primary Care Medicine Professionals on the Management of Comorbidities Related to Aging.   - Return 3.1. Medical visits to the haematology professional would be reduced, thanks to the fact that PC professionals could manage the comorbidities related to ageing in patients with HA over 65 years of age. - Return 3.4. The travel time of patients with HA over 65 to attend follow-up visits for their comorbidities would be reduced since visits would be to primary care instead of to the specialist. - Return 3.5. The burden on informal caregivers of HA patients older than 65 would be reduced, thanks to less time used to go with them to follow-up visits for their comorbidities. | | | |

Table 53. Sensitivity analysis. Assumption 9.

| Assumption included in the calculation (return) | Reference case | Worst-case scenario | Best-case scenario |
| --- | --- | --- | --- |
| Percentage of patients who would reduce their out-of-pocket expenses thanks to visits to primary care instead of specialized care. | 50% | 25% | 75% |
| Proposals and returns that include this assumption | | | |
| Proposal 3. Training for Primary Care Medicine Professionals on the Management of Comorbidities Related to Aging.   - Return 3.2. Out-of-pocket costs for HA patients over 65 years of age, related to travel to follow-up visits for comorbidities related to ageing, would be reduced. | | | |
|  | | | |

Table 54. Sensitivity analysis. Assumption 10.

| Assumption included in the calculation (return) | Reference case | Worst-case scenario | Best-case scenario |
| --- | --- | --- | --- |
| Percentage of patients older than 65 years who would improve their self-care and the scope of activities of daily living, by going to primary care instead of specialized care for the follow-up of their comorbidities. | 50% | 25% | 75% |
| Proposals and returns that include this assumption | | | |
| Proposal 3. Training for Primary Care Medicine Professionals on the Management of Comorbidities Related to Aging.   - Return 3.3. Self-care and day-to-day activities of patients with HA over 65 would improve | | | |

Table 55. Sensitivity analysis. Assumption 11.

| Assumption included in the calculation (return) | Reference case | Worst-case scenario | Best-case scenario |
| --- | --- | --- | --- |
| Percentage of patients with severe AH and inhibitor (prevalent). | 8.10% | 4.40% | 11.60% |
| Proposals and returns that include this assumption | | | |
| Proposal 4. Coordination between primary care health centres and haemophilia units through access to medical records throughout the national territory.   - Return 4.2. The pain of HA patients would be reduced, thanks to better coordination between levels of care. | | | |
| Proposal 7. Protocol for the treatment of pain and training for professionals related to the pathology.   - Return 7.1. The quality of life of patients with mild-moderate HA would be improved. - Return 7.2. The quality of life of patients with severe HA would be improved. - Return 7.3. The burden on informal caregivers of patients with HA would be reduced, thanks to better pain management. | | | |
| Proposal 11. Training for patients with inhibitor, and their families, on the management and care of haemophilia.   - Return 11.1 Medical visits to the hospital would be reduced, thanks to better management of the disease. - Return 11.2. The emotional state of patients with HA and inhibitor would be improved, thanks to better management of complications associated with the inhibitor. - Return 11.3. Trauma would be avoided in patients with prevalent HA and inhibitor, thanks to training in the prevention of those situations and behaviours that involve high risk. - Return 11.4. The mobility of patients with prevalent HA and inhibitor would be improved, thanks to better self-care and adherence after received training. - Return 11.7. The burden on informal caregivers of patients with prevalent HA and inhibitor would be reduced, thanks to the reduction in hospital visits. | | | |

Table 56. Sensitivity analysis. Assumption 12.

| Assumption included in the calculation (return) | Reference case | Worst-case scenario | Best-case scenario |
| --- | --- | --- | --- |
| Percentage of patients with mild-moderate AH and inhibitor (prevalent). | 1.55% | 0.64% | 2.46% |
| Proposals and returns that include this assumption | | | |
| Proposal 4. Coordination between primary care health centres and haemophilia units through access to medical records throughout the national territory.   - Return 4.2. The pain of HA patients would be reduced, thanks to better coordination between levels of care. | | | |
| Proposal 7. Protocol for the treatment of pain and training for professionals related to the pathology.   - Return 7.1. The quality of life of patients with mild-moderate HA would be improved. - Return 7.2. The quality of life of patients with severe HA would be improved. - Return 7.3. The burden on informal caregivers of patients with HA would be reduced, thanks to better pain management. | | | |
| Proposal 11. Training for patients with inhibitor, and their families, on the management and care of haemophilia   - Return 11.1 Medical visits to the hospital would be reduced, thanks to better management of the disease. - Return 11.2. The emotional state of patients with HA and inhibitor would be improved, thanks to better management of complications associated with the inhibitor. - Return 11.3. Trauma would be avoided in patients with prevalent HA and inhibitor, thanks to training in the prevention of those situations and behaviours that involve high risk. - Return 11.4. The mobility of patients with prevalent HA and inhibitor would be improved, thanks to better self-care and adherence after received training. - Return 11.7. The burden on informal caregivers of patients with prevalent HA and inhibitor would be reduced, thanks to the reduction in hospital visits. | | | |
| Proposal 12. Inhibitor eradication protocols.   - Return 12.6. The burden on informal caregivers of patients with HA and inhibitor would be reduced, thanks to the eradication of the inhibitor. | | | |

Table 57. Sensitivity analysis. Assumption 13.

| Assumption included in the calculation (return) | Reference case | Worst-case scenario | Best-case scenario |
| --- | --- | --- | --- |
| Average savings in healthcare resources per mild-moderate adult patient thanks to prior telematic consultation. | 9.58 € | 0.00 € | 19.17 € |
| Proposals and returns that include this assumption | | | |
| Proposal 5. Networking between small and reference centres, and between reference centres with each other   - Return 5.1. The referrals of adult patients with mild-moderate HA to other ACs would be reduced. | | | |

Table 58. Sensitivity analysis. Assumption 14.

| Assumption included in the calculation (return) | Reference case | Worst-case scenario | Best-case scenario |
| --- | --- | --- | --- |
| Average savings in healthcare resources per seriously ill adult and paediatric patient thanks to prior telematic consultation. | 12.82 € | 0.00 € | 25.65 € |
| Proposals and returns that include this assumption | | | |
| Proposal 5. Networking between small and reference centres, and between reference centres with each other.   - Return 5.2. Referrals of adult patients with severe HA and paediatric patients to other ACs would be reduced. | | | |

Table 59. Sensitivity analysis. Assumption 15.

| Assumption included in the calculation (return) | Reference case | Worst-case scenario | Best-case scenario |
| --- | --- | --- | --- |
| Number of kilometres of travel to a CSUR outside the ACs of residence. | 350 | 100 | 600 |
| Proposals and returns that include this assumption | | | |
| Proposal 5. Networking between small and reference centres, and between reference centres with each other   - Return 5.3. Referrals of patients with HA to other ACs to be seen by other specialists would be avoided. | | | |
| Proposal 9. Rapid referral protocol between autonomous communities to perform orthopaedic surgeries in reference centres   - Return 9.4. The out-of-pocket costs associated with travelling to other Autonomous Communities with the HA patient would increase. | | | |

Table 60. Sensitivity analysis. Assumption 16.

| Assumption included in the calculation (return) | Reference case | Worst-case scenario | Best-case scenario |
| --- | --- | --- | --- |
| Time (hours) used in a face-to-face consultation with other ACs. | 6 | 8 | 4 |
| Proposals and returns that include this assumption | | | |
| Proposal 5. Networking between small and reference centres, and between reference centres with each other   - Return 5.4. Losses of labour productivity of patients with HA who work would be reduced. - Return 5.6. The burden of informal caregivers of patients with HA would be reduced. | | | |

Table 61. Sensitivity analysis. Assumption 17.

| Assumption included in the calculation (return) | Reference case | Worst-case scenario | Best-case scenario |
| --- | --- | --- | --- |
| Percentage of patients who would improve their satisfaction thanks to the networking. | 20% | 10% | 30% |
| Proposals and returns that include this assumption | | | |
| Proposal 5. Networking between small and reference centres, and between reference centres with each other.   - Return 5.5. Adult patients with HA would be more satisfied with the NHS. - Return 5.7. Informal caregivers of patients with HA would be more satisfied with the NHS. | | | |

Table 62. Sensitivity analysis. Assumption 18.

| Assumption included in the calculation (return) | Reference case | Worst-case scenario | Best-case scenario |
| --- | --- | --- | --- |
| Percentage of patients with mild-moderate AH and inhibitor (incident). | 4.65% | 3.60% | 5.30% |
| Proposals and returns that include this assumption | | | |
| Proposal 7. Protocol for the treatment of pain and training for professionals related to the pathology.   - Return 7.1. The quality of life of patients with mild-moderate HA would be improved. - Return 7.2. The quality of life of patients with severe HA would be improved. - Return 7.3. The burden on informal caregivers of patients with HA would be reduced, thanks to better pain management. | | | |
| Proposal 11. Training for patients with inhibitor, and their families, on the management and care of haemophilia   - Return 11.2. The emotional state of patients with HA and inhibitor would be improved, thanks to better management of complications associated with the inhibitor. - Return 11.5. The emotional state (stress) of the informal caregivers of patients with incidental HA and inhibitor would be improved. - Return 11.6. The burden of informal caregivers of patients with incidental HA and inhibitor would increase when accompanying them to face-to-face training workshops. | | | |
| Proposal 12. Inhibitor eradication protocols.   - Return 12.2. The mobility of patients with HA and inhibitor would be improved, thanks to better joint health after the eradication of the inhibitor. - Return 12.3. The pain and discomfort of patients with HA and inhibitor would be reduced, thanks to more successful inhibitor eradication. - Return 12.4. Improvement of day-to-day activities performance of patients with HA and inhibitor, thanks to inhibitor eradication. - Return 12.5. The time needed by patients with incidental HA and inhibitor to comply with the inhibitor eradication treatment (more visits, more frequent doses) would increase. - Return 12.6. The burden on informal caregivers of patients with HA and inhibitor would be reduced, thanks to the eradication of the inhibitor. - Return 12.7. The burden on informal caregivers of patients with incidental HA and inhibitor would increase due to the increase in the time required to comply with the inhibitor eradication treatment (more visits, more frequent doses). | | | |

Table 63. Sensitivity analysis. Assumption 19.

| Assumption included in the calculation (return) | Reference case | Worst-case scenario | Best-case scenario |
| --- | --- | --- | --- |
| Percentage of patients with incidental severe AH and inhibitor. | 30% | 25% | 35% |
| Proposals and returns that include this assumption | | | |
| Proposal 11. Training for patients with inhibitor, and their families, on the management and care of haemophilia   - Return 11.2. The emotional state of patients with HA and inhibitor would be improved, thanks to better management of complications associated with the inhibitor. - Return 11.5. The emotional state (stress) of the informal caregivers of patients with incidental HA and inhibitor would be improved. - Return 11.6. The burden of informal caregivers of patients with incidental HA and inhibitor would increase when accompanying them to face-to-face training workshops. | | | |
| Proposal 12. Inhibitor eradication protocols.   - Return 12.1. The direct healthcare costs related to the treatment of patients with severe HA would be reduced, thanks to greater success in the inhibitor eradication - Return 12.2. The mobility of patients with HA and inhibitor would be improved, thanks to better joint health after the eradication of the inhibitor. - Return 12.3. The pain and discomfort of patients with HA and inhibitor would be reduced, thanks to more successful inhibitor eradication. - Return 12.4. Improvement of day-to-day activities performance of patients with HA and inhibitor, thanks to inhibitor eradication. - Return 12.5. The time needed by patients with incidental HA and inhibitor to comply with the inhibitor eradication treatment (more visits, more frequent doses) would increase. - Return 12.6. The burden on informal caregivers of patients with HA and inhibitor would be reduced, thanks to the eradication of the inhibitor. - Return 12.7. The burden on informal caregivers of patients with incidental HA and inhibitor would increase due to the increase in the time required to comply with the inhibitor eradication treatment (more visits, more frequent doses). | | | |

Table 64. Sensitivity analysis. Assumption 20.

| Assumption included in the calculation (return) | Reference case | Worst-case scenario | Best-case scenario |
| --- | --- | --- | --- |
| Percentage of patients with AH in whom the development of haemophilic arthropathy would be avoided. | 50% | 25% | 75% |
| Proposals and returns that include this assumption | | | |
| Proposal 8. Early Diagnosis of Arthropathy to Adapt Prophylaxis.   - Return 8.1. The healthcare costs associated with the management of haemophilic arthropathy in patients with severe HA would be reduced. - Return 8.3. The functional status and quality of life of patients with severe HA would be maintained by avoiding the development of haemophilic arthropathy. | | | |

Table 65. Sensitivity analysis. Assumption 21.

| Assumption included in the calculation (return) | Reference case | Worst-case scenario | Best-case scenario |
| --- | --- | --- | --- |
| Number of days of hospitalization avoided when undergoing orthopaedic surgery in a CSUR. | 1.47 | 0.00 | 2.94 |
| Proposals and returns that include this assumption | | | |
| Proposal 9. Rapid referral protocol between autonomous communities to perform orthopaedic surgeries in reference centres.   - Return 9.2. Healthcare costs would be reduced thanks to fewer days of hospitalization for orthopaedic surgery in patients with moderate-severe HA. | | | |

Table 66. Sensitivity analysis. Assumption 22.

| Assumption included in the calculation (return) | Reference case | Worst-case scenario | Best-case scenario |
| --- | --- | --- | --- |
| Average number of medical visits in the hospital setting per year for patients with prevalent HA and inhibitor. | 4 | 2 | 6 |
| Proposals and returns that include this assumption | | | |
| Proposal 11. Training for patients with inhibitor, and their families, on the management and care of haemophilia.   - Return 11.1 Medical visits to the hospital would be reduced, thanks to better management of the disease. - Return 11.7. The burden on informal caregivers of patients with prevalent HA and inhibitor would be reduced, thanks to the reduction in hospital visits. | | | |

Table 67. Sensitivity analysis. Assumption 23.

| Assumption included in the calculation (return) | Reference case | Worst-case scenario | Best-case scenario |
| --- | --- | --- | --- |
| Percentage of medical visits in the hospital setting per year that could be reduced thanks to training for patients with inhibitors and their informal caregivers. | 50% | 25% | 75% |
| Proposals and returns that include this assumption | | | |
| Proposal 11. Training for patients with inhibitor, and their families, on the management and care of haemophilia.   - Return 11.1 Medical visits to the hospital would be reduced, thanks to better management of the disease. - Return 11.7. The burden on informal caregivers of patients with prevalent HA and inhibitor would be reduced, thanks to the reduction in hospital visits. | | | |

Table 68. Sensitivity analysis. Assumption 24.

| Assumption included in the calculation (return) | Reference case | Worst-case scenario | Best-case scenario |
| --- | --- | --- | --- |
| Average cost of treating mild-moderate bleeding. | €8,852.10 | €6,908.48 | €13,387.19 |
| Proposals and returns that include this assumption | | | |
| Proposal 11. Training for patients with inhibitor, and their families, on the management and care of haemophilia.   - Return 11.3. Trauma would be avoided in patients with prevalent HA and inhibitor, thanks to training in the prevention of those situations and behaviours that involve high risk. | | | |

Table 69. Sensitivity analysis. Assumption 25.

| Assumption included in the calculation (return) | Reference case | Worst-case scenario | Best-case scenario |
| --- | --- | --- | --- |
| Success rate of induction of immune tolerance treatment, after the implementation of the eradication protocol. | 78% | 76% | 81% |
| Proposals and returns that include this assumption | | | |
| Proposal 12. Inhibitor eradication protocols.   - Return 12.1. The direct healthcare costs related to the treatment of patients with severe HA would be reduced, thanks to greater success in the inhibitor eradication - Return 12.2. The mobility of patients with HA and inhibitor would be improved, thanks to better joint health after the eradication of the inhibitor. - Return 12.3. The pain and discomfort of patients with HA and inhibitor would be reduced, thanks to more successful inhibitor eradication. - Return 12.4. Improvement of day-to-day activities performance of patients with HA and inhibitor, thanks to inhibitor eradication. - Return 12.5. The time needed by patients with incidental HA and inhibitor to comply with the inhibitor eradication treatment (more visits, more frequent doses) would increase. - Return 12.6. The burden on informal caregivers of patients with HA and inhibitor would be reduced, thanks to the eradication of the inhibitor. - Return 12.7. The burden on informal caregivers of patients with incidental HA and inhibitor would increase due to the increase in the time required to comply with the inhibitor eradication treatment (more visits, more frequent doses). | | | |

Table 70. Sensitivity analysis. Assumption 26.

| Assumption included in the calculation (return) | Reference case | Worst-case scenario | Best-case scenario |
| --- | --- | --- | --- |
| Percentage of hours of informal care that would be reduced, thanks to the eradication of the inhibitor. | 25% | 15% | 35% |
| Proposals and returns that include this assumption | | | |
| Proposal 12. Inhibitor eradication protocols.   - Return 12.6. The burden on informal caregivers of patients with HA and inhibitor would be reduced, thanks to the eradication of the inhibitor. | | | |

Table 71. Sensitivity analysis. Assumption 27.

| Assumption included in the calculation (return) | Reference case | Worst-case scenario | Best-case scenario |
| --- | --- | --- | --- |
| Percentage of hours of informal care that would increase, due to the necessary time for compliance with the treatment for the eradication of the inhibitor. | 25% | 35% | 15% |
| Proposals and returns that include this assumption | | | |
| Proposal 12. Inhibitor eradication protocols.   - Return 12.7. The burden on informal caregivers of patients with incidental HA and inhibitor would increase due to the increase in the time required to comply with the inhibitor eradication treatment (more visits, more frequent doses). | | | |

Table 72. Sensitivity analysis. Assumption 28.

| Assumption included in the calculation (return) | Reference case | Worst-case scenario | Best-case scenario |
| --- | --- | --- | --- |
| Average of teaching hours per day. | 5 | 4 | 6 |
| Proposals and returns that include this assumption | | | |
| Proposal 14. Training for education professionals about the care of students with haemophilia in schools   - Return 14.1. School delays in paediatric HA patients due to missed school hours would be avoided, thanks to the training of education professionals in the management of the student with HA. | | | |
| Proposal 15. Training for paediatric patients on adherence to treatment.   - Return 15.4. The days of school missed by paediatric HA patients would be reduced, thanks to a better physical condition and greater control of the pathology. | | | |

Table 73. Sensitivity analysis. Assumption 29.

| Assumption included in the calculation (return) | Reference case | Worst-case scenario | Best-case scenario |
| --- | --- | --- | --- |
| Percentage of teachers trained in the management of students with HA. | 10% | 20% | 0% |
| Proposals and returns that include this assumption | | | |
| Proposal 14. Training for education professionals about the care of students with haemophilia in schools   - Return 14.1. School delays in paediatric HA patients due to missed school hours would be avoided, thanks to the training of education professionals in the management of the student with HA. - Return 14.2. The social relationships of paediatric HA patients would be improved, thanks to better school integration. - Return 14.3. The physical activity of students with HA would be improved, thanks to more participation in school sports activities. - Return 14.4. The emotional state of the informal caregivers of paediatric HA patients would be improved, thanks to the correct management of their children’s disease in schools. - Return 14.5. The burden on informal caregivers of paediatric HA patients would be reduced. | | | |

Table 74. Sensitivity analysis. Assumption 30.

| Assumption included in the calculation (return) | Reference case | Worst-case scenario | Best-case scenario |
| --- | --- | --- | --- |
| Success rate of the training to increase adherence. | 50% | 25% | 75% |
| Proposals and returns that include this assumption | | | |
| Proposal 15. Training for paediatric patients on adherence to treatment.   - Return 15.1. The physical condition of paediatric HA patients would be improved and pain would be reduced, thanks to increased adherence. - Return 15.2. The mood of paediatric HA patients would improve, thanks to a better physical condition and greater control of the disease. - Return 15.3. The social relationships of paediatric HA patients would be improved, thanks to a better physical condition and greater control of the disease. - Return 15.4. The days of school missed by paediatric HA patients would be reduced, thanks to a better physical condition and greater control of the pathology. - Return 15.5. The emotional state of informal caregivers of paediatric HA patients would be improved, thanks to better management of the pathology. - Return 15.6. The burden on informal caregivers of paediatric HA patients would be reduced, thanks to better disease control. - Return 15.7. Family dynamics would improve, thanks to better adherence. | | | |

Table 75. Sensitivity analysis. Assumption 31.

| Assumption included in the calculation (return) | Reference case | Worst-case scenario | Best-case scenario |
| --- | --- | --- | --- |
| Number of hours of informal care per day when the paediatric HA patient is unwell. | 12 | 8 | 16 |
| Proposals and returns that include this assumption | | | |
| Proposal 15. Training for paediatric patients on adherence to treatment.   - Return 15.6. The burden on informal caregivers of paediatric HA patients would be reduced, thanks to better disease control. | | | |

# Table Index

[Table 1. Proposals for improving the approach to Haemophilia A (HA). General area. 4](#_Toc83316176)

[Table 2. Proposals for improving the approach to HA. Area of patients with arthropathy. 6](#_Toc83316177)

[Table 3. Proposals for improving the approach to HA. Area of patients with inhibitors. 8](#_Toc83316178)

[Table 4. Proposals for improving the approach to HA. Area of paediatric patients. 10](#_Toc83316179)

[Table 5. Proposal 1 Investment Breakdown: Attention by multidisciplinary teams with all specialities involved. 12](#_Toc83316180)

[Table 6. Proposal 2 Investment Breakdown: Nursing hospital case manager. 15](#_Toc83316181)

[Table 7. Proposal 3 Investment Breakdown: Training for Primary Care Medicine Professionals on the Management of Comorbidities Related to Aging. 16](#_Toc83316182)

[Table 8. Proposal 4 Investment Breakdown: Coordination between primary care health centres and haemophilia units through access to medical records throughout the national territory. 17](#_Toc83316183)

[Table 9. Proposal 5 Investment Breakdown: Networking between small and reference centres, and between reference centres with each other. 17](#_Toc83316184)

[Table 10. Proposal 6 Investment Breakdown: Bringing hospital medication closer to patients. 18](#_Toc83316185)

[Table 11. Proposal 7 Investment Breakdown: Protocol for pain treatment and training for professionals related to the pathology. 19](#_Toc83316186)

[Table 12. Proposal 8 Investment Breakdown: Early Diagnosis of Arthropathy to Adapt Prophylaxis. 20](#_Toc83316187)

[Table 13. Proposal 9 Investment Breakdown: Rapid referral protocol between autonomous communities to perform orthopaedic surgeries in reference centres. 20](#_Toc83316188)

[Table 14. Proposal 10 Investment Breakdown: Development of a patient national registry. 21](#_Toc83316189)

[Table 15. Proposal 11 Investment Breakdown: Training for patients with inhibitor, and their families, on the management and care of haemophilia. 22](#_Toc83316190)

[Table 16. Proposal 12 Investment Breakdown: Inhibitor eradication protocols. 23](#_Toc83316191)

[Table 17. Proposal 13 Investment Breakdown: Training for parents on the management of haemophilia, in health centres and patient groups. 24](#_Toc83316192)

[Table 18. Proposal 14 Investment Breakdown: Training for education professionals about the care of students with haemophilia in schools. 25](#_Toc83316193)

[Table 19. Proposal 15 Investment Breakdown: Training for paediatric patients on adherence to treatment. 26](#_Toc83316194)

[Table 20. Proposal 1 Return Breakdown: Attention by multidisciplinary teams with all specialities involved. 27](#_Toc83316195)

[Table 21. Proposal 2 Return Breakdown: hospital case manager nurse. 30](#_Toc83316196)

[Table 22. Proposal 3 Return Breakdown: Training for Primary Care Medicine Professionals on the Management of Comorbidities Related to Aging. 32](#_Toc83316197)

[Table 23. Proposal 4 Return Breakdown: Coordination between primary care health centres and haemophilia units through access to medical records throughout the national territory 34](#_Toc83316198)

[Table 24. Proposal 5 Return Breakdown: Networking between small and reference centres, and between reference centres with each other. 35](#_Toc83316199)

[Table 25. Proposal 6 Return Breakdown: Bringing hospital medication closer to patients. 37](#_Toc83316200)

[Table 26. Proposal 7 Return Breakdown: Protocol for the treatment of pain and training for professionals related to the pathology. 38](#_Toc83316201)

[Table 27. Proposal 8 Return Breakdown: Early Diagnosis of Arthropathy to Adapt Prophylaxis. 38](#_Toc83316202)

[Table 28. Proposal 9 Return Breakdown: Rapid referral protocol between autonomous communities to perform orthopaedic surgeries in reference centres 40](#_Toc83316203)

[Table 29. Proposal 11 Return Breakdown: Training for patients with inhibitor, and their families, on the management and care of haemophilia 42](#_Toc83316204)

[Table 30. Proposal 12 Return Breakdown: Inhibitor eradication protocols. 43](#_Toc83316205)

[Table 31. Proposal 13 Return Breakdown: Training for parents on the management of haemophilia, in health centres and patient groups. 46](#_Toc83316206)

[Table 32. Proposal 14 Return Breakdown: Training for education professionals about the care of students with haemophilia in schools 48](#_Toc83316207)

[Table 33. Proposal 15 Return Breakdown: Training for paediatric patients on adherence to treatment. 50](#_Toc83316208)

[Table 34. Assumptions of Proposal 1. Attention from multidisciplinary teams with all specialities involved. 53](#_Toc83316209)

[Table 35. Assumptions of Proposal 2. Hospital case manager nurse. 53](#_Toc83316210)

[Table 36. Assumptions of Proposal 3. Training for primary care medical professionals on the management of ageing-related comorbidities. 53](#_Toc83316211)

[Table 37. Assumptions of Proposal 5. Networking between small and reference centres, and between reference centres with each other 54](#_Toc83316212)

[Table 38. Assumptions of Proposal 7. Protocol for pain treatment and training for professionals related to the pathology 54](#_Toc83316213)

[Table 39. Assumptions of Proposal 8. Early diagnosis of arthropathy to adapt prophylaxis 54](#_Toc83316214)

[Table 40. Assumptions of Proposal 10. Development of a patient national registry 54](#_Toc83316215)

[Table 41. Assumptions of Proposal 11 Training for patients with inhibitor, and their families, on the management and care of haemophilia 55](#_Toc83316216)

[Table 42. Assumptions of Proposal 13. Training for parents on the management of haemophilia, delivered in health centres and patient groups 55](#_Toc83316217)

[Table 43. Assumptions of Proposal 14. Training for education professionals about the care of students with haemophilia in schools. 55](#_Toc83316218)

[Table 44. Assumptions of Proposal 15. Training for paediatric patients on adherence to treatment 56](#_Toc83316219)

[Table 45. Sensitivity analysis. Assumption 1. 56](#_Toc83316220)

[Table 46. Sensitivity analysis. Assumption 2. 56](#_Toc83316221)

[Table 47. Sensitivity analysis. Assumption 3. 57](#_Toc83316222)

[Table 48. Sensitivity analysis. Assumption 4. 57](#_Toc83316223)

[Table 49. Sensitivity analysis. Assumption 5. 58](#_Toc83316224)

[Table 50. Sensitivity analysis. Assumption 6. 58](#_Toc83316225)

[Table 51. Sensitivity analysis. Assumption 7. 59](#_Toc83316226)

[Table 52. Sensitivity analysis. Assumption 8. 59](#_Toc83316227)

[Table 53. Sensitivity analysis. Assumption 9. 60](#_Toc83316228)

[Table 54. Sensitivity analysis. Assumption 10. 60](#_Toc83316229)

[Table 55. Sensitivity analysis. Assumption 11. 60](#_Toc83316230)

[Table 56. Sensitivity analysis. Assumption 12. 61](#_Toc83316231)

[Table 57. Sensitivity analysis. Assumption 13. 62](#_Toc83316232)

[Table 58. Sensitivity analysis. Assumption 14. 62](#_Toc83316233)

[Table 59. Sensitivity analysis. Assumption 15. 62](#_Toc83316234)

[Table 60. Sensitivity analysis. Assumption 16. 63](#_Toc83316235)

[Table 61. Sensitivity analysis. Assumption 17. 63](#_Toc83316236)

[Table 62. Sensitivity analysis. Assumption 18. 63](#_Toc83316237)

[Table 63. Sensitivity analysis. Assumption 19. 64](#_Toc83316238)

[Table 64. Sensitivity analysis. Assumption 20. 65](#_Toc83316239)

[Table 65. Sensitivity analysis. Assumption 21. 65](#_Toc83316240)

[Table 66. Sensitivity analysis. Assumption 22. 65](#_Toc83316241)

[Table 67. Sensitivity analysis. Assumption 23. 66](#_Toc83316242)

[Table 68. Sensitivity analysis. Assumption 24. 66](#_Toc83316243)

[Table 69. Sensitivity analysis. Assumption 25. 66](#_Toc83316244)

[Table 70. Sensitivity analysis. Assumption 26. 67](#_Toc83316245)

[Table 71. Sensitivity analysis. Assumption 27. 67](#_Toc83316246)

[Table 72. Sensitivity analysis. Assumption 28. 67](#_Toc83316247)

[Table 73. Sensitivity analysis. Assumption 29. 68](#_Toc83316248)

[Table 74. Sensitivity analysis. Assumption 30. 68](#_Toc83316249)

[Table 75. Sensitivity analysis. Assumption 31. 69](#_Toc83316250)

# References

1. Ministerio de Sanidad, Servicios Sociales e Igualdad. Hemofilia. Aspectos Organizativos. 2012.

2. Nugent D, Kalnins W, Querol F, Gregory M, Pilgaard T, Cooper DL, et al. Haemophilia Experiences, Results and Opportunities (HERO) study: treatment-related characteristics of the population. Haemophilia. 2015;21(1):e26-38, doi: 10.1111/hae.12545.

3. Srivastava A, Brewer AK, Mauser-Bunschoten EP, Key NS, Kitchen S, Llinas A, et al. Guidelines for the management of haemophilia. Haemophilia. 2013;19(1):e1-47, doi: 10.1111/j.1365-2516.2012.02909.x.

4. Mediana de las tarifas sanitarias oficiales de las comunidades autónomas. 2019.

5. De la Corte-Rodriguez H, Rodriguez-Merchan EC, Alvarez-Roman MT, Martin-Salces M, Martinoli C, Jimenez-Yuste V. The value of HEAD-US system in detecting subclinical abnormalities in joints of patients with haemophilia. Expert Rev Hematol. 2018;11(3):253-61, doi: 10.1080/17474086.2018.1435269.

6. Proyecto SROI-HA. Asunción. 2020.

7. Al‐Huniti A, Reyes Hernandez M, Ten Eyck P, Staber JM. Mental health disorders in haemophilia: Systematic literature review and meta‐analysis. Haemophilia. 2020;26(3):431-42, doi: 10.1111/hae.13960.

8. Wilding J, Zourikian N, Minno MD, Khair K, Marquardt N, Benson G, et al. Obesity in the global haemophilia population: prevalence, implications and expert opinions for weight management. Obes Rev. 2018;19(11):1569-84, doi: 10.1111/obr.12746.

9. Universitat de Valencia. Master en Enfermedades Raras a distancia. 2020, Disponible en: https://postgrado.adeituv.es/es/cursos/area_de_salud-7/enfermedades-raras/datos_generales.htm.

10. Federación Española de Hemofilia. Centros de Tratamiento de Hemofilia. Fedhemo. 2020, Disponible en: http://fedhemo.com/centros-de-tratamiento/.

11. Schrijvers L, Bedford M, Elfvinge P, Andritschke K, Leenders B, Harrington C. The role of the European haemophilia nurse. J Haemoph Pract. 2014;1(1):24-7, doi: 10.17225/jhp.00008.

12. Proyecto SROI-HA. Precio de mercado. 2020.

13. Consejería de Sanidad. Comunidad de Madrid. Comisión de Formación Continuada de las Profesiones Sanitarias. Instrucciones y Normas de Tramitación de las Solicitudes de Acreditación de Actividades de Formación Continuada de las Profesiones Sanitarias en la Comunidad de Madrid. 2017.

14. Sociedad Española de Hematología y Hemoterapia. Sociedad Española de Hematología y Hemoterápia - Aval científico. [accedido 5 junio 2020]. Disponible en: https://www.sehh.es/avales/cientifico.

15. Sociedad Española de Trombosis y Hemostasia. Reglamento solicitud de auspicios SETH. Sociedad Española de Trombosis y Hemostasia. [accedido 8 junio 2020]. Disponible en: https://www.seth.es/index.php/seth/la-sociedad/reglamento-de-auspicios-seth.html.

16. Sociedad Española de Médicos de Atención Primaria. Manual de procedimientos. 2014.

17. Organización Médica Colegial: Fundación para la formación. REGLAMENTO DE CURSOS Y ACCIONES FORMATIVAS. [accedido 22 marzo 2018]. Disponible en: http://www.ffomc.org/sites/default/files/ReglamentoVersionFinal.pdf.

18. Sociedad Española de Informática de la Salud. Índice SEIS 2019. 2020.

19. Lucía JF, Aznar JA, Abad-Franch L, Escuin RR, Jiménez-Yuste V, Pérez R, et al. Prophylaxis therapy in haemophilia A: current situation in Spain. Haemophilia. 2011;17(1):75-80, doi: 10.1111/j.1365-2516.2010.02378.x.

20. Megías-Vericat JE, Monte-Boquet E, Martín-Cerezuela M, Cuéllar-Monreal MJ, Tarazona-Casany MV, Pérez-Huertas P, et al. Pilot evaluation of home delivery programme in haemophilia. J Clin Pharm Ther. 2018;43(6):822-8, doi: 10.1111/jcpt.12718.

21. Fedhemo. Home Delivery. Fedhemo. s. f., Disponible en: http://fedhemo.com/home-delivery/.

22. Sociedad Española de Hematología y Hemoterapia, Sociedad Española de Trombosis y Hemostasia. Inscripción al Congreso de la SEHH y SETH. SEHH SETH. [accedido 25 mayo 2020]. Disponible en: https://www.sehhseth.es/informacion/inscripcion.

23. Servicio Madrileño de Salud. Guía para la elaboración de protocolos y procedimientos enfermeros. 2012.

24. Cuesta-Barriuso R, Torres-Ortuño A, López-García M, Nieto-Munuera J. Effectiveness of an educational intervention of physiotherapy in parents of children with haemophilia. Haemoph Off J World Fed Hemoph. 2014;20(6):866-72, doi: 10.1111/hae.12447.

25. Federación Española de Hemofilia. Asociaciones de Hemofilia en España. Fedhemo. 2015, Disponible en: http://fedhemo.com/asociaciones/.

26. Instituto Nacional de Estadística. Estadística del Padrón continuo. Población por provincias, edad (año a año), Españoles/Extranjeros, Sexo y Año. INE. [accedido 24 junio 2020]. Disponible en: https://www.ine.es/jaxi/Datos.htm?path=/t20/e245/p08/l0/&file=03003.px#!tabs-tabla.

27. Martín-Fernández J, Rodríguez-Martínez G, Ariza-Cardiel G, Vergel Gutierrez MÁ, Hidalgo Escudero AV, Conde-López JF. Variables que condicionan la utilización de la consulta de enfermería en centros de salud de la Comunidad de Madrid. Rev Esp Salud Pública. 2013;87(4):383-92, doi: 10.4321/S1135-57272013000400008.

28. Pai M, Key NS, Skinner M, Curtis R, Feinstein M, Kessler C, et al. NHF-McMaster Guideline on Care Models for Haemophilia Management. Haemophilia. 2016;22:6-16, doi: 10.1111/hae.13008.

29. Instituto para el Desarrollo e Integración de la Sanidad. Sanidad privada, aportando valor. Análisis de Situación 2019. 2019.

30. Righolt AJ, Jevdjevic M, Marcenes W, Listl S. Global-, Regional-, and Country-Level Economic Impacts of Dental Diseases in 2015. J Dent Res. 2018;97(5):501-7, doi: 10.1177/0022034517750572.

31. Hoxer CS, Zak M, BenmedjHAed K, Lambert J. Utility valuation of health states for haemophilia and related complications in Europe and in the United States. Haemophilia. 2019;25(1):92-100, doi: 10.1111/hae.13634.

32. Ortega Eslava A, Marín Gil R, Fraga Fuentes MD, López-Briz E, Puigventós Latorre F. Guía de evaluación económica e impacto presupuestario en los informes de evaluación de medicamentos. SEFH. Sociedad Española de Farmacia Hospitalaria; 2016.

33. O’Hara J, Walsh S, Camp C, Mazza G, Carroll L, Hoxer C, et al. The impact of severe haemophilia and the presence of target joints on health-related quality-of-life. Health Qual Life Outcomes. 2018;16(1):84, doi: 10.1186/s12955-018-0908-9.

34. Hashim M, Franks P, Fiscella K. Effectiveness of Telephone Reminders in Improving Rate of Appointments Kept at an Outpatient Clinic: A Randomized Controlled Trial. J Am Board Fam Pract. 2001;14(3):193-6.

35. Instituto Nacional de Estadística. Encuesta Anual de Estructura Salarial 2016. Resultados Nacionales y por Comunidades Autónomas. Ganancia por hora normal de trabajo. [accedido 21 diciembre 2018]. Disponible en: https://www.ine.es/jaxiT3/Datos.htm?t=28205.

36. BOE Num 312. España. Real Decreto 1462/2018, de 21 de diciembre, por el que se fija el salario mínimo interprofesional para 2019. BOE núm. 312, de 27 de diciembre de 2018. 2019:5.

37. Angelini D, Konkle BA, Sood SL. Aging among persons with haemophilia: contemporary concerns. Semin Hematol. 2016;53(1):35-9, doi: 10.1053/j.seminhematol.2015.10.004.

38. Ministerio de Sanidad, Consumo y Bienestar Social. Consulta Interactiva del SNS. Base de Datos de Clínicos de Atención Primaria (BDCAP). [accedido 17 junio 2020]. Disponible en: https://pestadistico.inteligenciadegestion.mscbs.es/publicoSNS/Comun/ArbolNodos.aspx?idNodo=22117.

39. Cavazza M, Kodra Y, Armeni P, De Santis M, López-Bastida J, Linertová R, et al. Social/economic costs and quality of life in patients with haemophilia in Europe. Eur J Health Econ HEPAC Health Econ Prev Care. 2016;17 Suppl 1:53-65, doi: 10.1007/s10198-016-0785-2.

40. Instituto Nacional de Estadística. Encuesta de presupuestos familiares. Base 2006. Gasto por grupos de gasto (2 dígitos). Gasto medio por persona y año en ocio y cultura. INE. [accedido 15 junio 2020]. Disponible en: https://www.ine.es/jaxiT3/Tabla.htm?t=24765&L=0.

41. Instituto Nacional de Estadística. INEbase. Servicios Hostelería y turismo. Encuesta de turismo de residentes. Series trimestrales. Resultados nacionales. Viajes, pernoctaciones, duración media y gasto por tipo de destino principal (acumulado 2019T4). INE. [accedido 12 junio 2020]. Disponible en: https://www.ine.es/jaxiT3/Datos.htm?t=12427#!tabs-tabla.

42. Ministerio de Sanidad. Sistema HCDSNS. Historia Clínica Digital del Sistema Nacional de Salud. Informe de situación 1 de abril de 2020. 2020.

43. Aznar JA, Altisent C, Álvarez-Román MT, Bonanad S, Mingot-Castellano ME, López MF. Moderate and severe haemophilia in Spain: An epidemiological update. Haemophilia. 2018;24(3):e136-9, doi: 10.1111/hae.13462.

44. Morfini M, Haya S, Tagariello G, Pollmann H, Quintana M, Siegmund B, et al. European study on orthopaedic status of haemophilia patients with inhibitors. Haemoph Off J World Fed Hemoph. 2007;13(5):606-12, doi: 10.1111/j.1365-2516.2007.01518.x.

45. Ministerio de Sanidad, Consumo y Bienestar Social. Barómetro Sanitario 2018. 2018.

46. España. ORDEN EHA/3770/2005, de 1 de diciembre, por la que se revisa el importe de la indemnización por uso de vehículo particular establecida en el Real Decreto 462/2002, de 24 de mayo, sobre indemnizaciones por razón del servicio. 2005.

47. Margusino-Framiñán L. Implantación de Consultas Externas Monográficas de Atención Farmacéutica en un Servicio de. Farm Hosp. 2017;(06):660-9, doi: 10.7399/fh.10771.

48. van Hout B, Janssen M, Feng Y-S, Kohlmann T, Busschbach J, Golicki D, et al. Interim scoring for the EQ-5D-5L: Mapping the EQ-5D-5L to EQ-5D-3L value sets. Value Health. 2012;15(5):708-15, doi: 10.1016/j.jval.2012.02.008.

49. O’Hara J, Walsh S, Camp C, Mazza G, Carroll L, Hoxer C, et al. The relationship between target joints and direct resource use in severe haemophilia. Health Econ Rev. 2018;8(1):1-7, doi: 10.1186/s13561-018-0185-7.

50. Moore MF, Tobase P, Allen DD. Meta-analysis: outcomes of total knee arthroplasty in the haemophilia population. Haemophilia. 2016;22(4):e275-85, doi: 10.1111/hae.12885.

51. Santos Silva M, Rodrigues-Pinto R, Rodrigues C, Morais S, Costa e Castro J. Long-term results of total knee arthroplasty in hemophilic arthropathy. J Orthop Surg. 2019;27(1):230949901983433, doi: 10.1177/2309499019834337.

52. Ministerio de Sanidad, Consumo y Bienestar Social. Consulta Interactiva del SNS. Registro de Actividad de Atención Especializada - RAE-CMBD (Coste medio por malfuncionamiento, reacción o complicación de dispositivo o procedimiento ortopédico.). [accedido 20 junio 2020]. Disponible en: https://pestadistico.inteligenciadegestion.mscbs.es/publicoSNS/Comun/Cubo.aspx?IdNodo=23619.

53. Ministerio de Sanidad, Consumo y Bienestar Social. Consulta Interactiva del SNS. Registro de Actividad de Atención Especializada - RAE-CMBD (Coste medio por la sustitución de articulación de rodilla). [accedido 22 junio 2020]. Disponible en: https://pestadistico.inteligenciadegestion.mscbs.es/publicoSNS/Comun/Cubo.aspx?IdNodo=23619.

54. Ministerio de Sanidad, Consumo y Bienestar Social. Consulta Interactiva del SNS. Registro de Actividad de Atención Especializada - RAE-CMBD (Estancia media días (hospitalización). [accedido 22 junio 2020]. Disponible en: https://pestadistico.inteligenciadegestion.mscbs.es/publicoSNS/Comun/Cubo.aspx?IdNodo=23619.

55. Instituto Nacional de Estadística. Encuesta de turismo de residentes. Series anuales. Viajes, pernoctaciones, duración media y gasto por alojamiento principal, según tipo de destino principal. INE. [accedido 24 junio 2020]. Disponible en: https://www.ine.es/jaxiT3/Datos.htm?t=24920#!tabs-tabla.

56. De la Corte-Rodriguez H, Rodriguez-Merchan EC, Alvarez-Roman T, Martin-Salces M, Garcia-Barcenilla S, Jimenez-Yuste V. Health education and empowerment in adult patients with haemophilia. Expert Rev Hematol. 2019;12(11):989-95, doi: 10.1080/17474086.2019.1650640.

57. Proyecto SROI-HA. Comité Asesor. 2020.

58. Morado M, Villar A, Jiménez Yuste V, Quintana M, Hernandez Navarro F. Prophylactic treatment effects on inhibitor risk: experience in one centre. Haemoph Off J World Fed Hemoph. 2005;11(2):79-83, doi: 10.1111/j.1365-2516.2005.00921.x.

59. Mulders G, de Wee EM, VHAedi Nikbakht-Van de Sande MCVM, Kruip MJHA, Elfrink EJ, Leebeek FWG. E-learning improves knowledge and practical skills in haemophilia patients on home treatment: a randomized controlled trial. Haemoph Off J World Fed Hemoph. 2012;18(5):693-8, doi: 10.1111/j.1365-2516.2012.02786.x.

60. Rehm H, Schmolders J, Koob S, Bornemann R, Goldmann G, Oldenburg J, et al. Falling and fall risk in adult patients with severe haemophilia. Hamostaseologie. 2017;37(2):97-103, doi: 10.5482/HAMO-16-03-0009.

61. Jiménez-Yuste V, Álvarez Román MT, Mingot-Castellano ME, Fernández Mosteirin N, Mareque M, Oyagüez I. Análisis de costes del tratamiento para pacientes con hemofilia A con inhibidor en España. PharmacoEconomics Span Res Artic. 2018;15(1):25-34, doi: 10.1007/s40277-018-0080-y.

62. Ministerio de Sanidad, Consumo y Bienestar Social. Orden SCB/45/2019, de 22 de enero por el que se establece la cartera de servicios comunes del Sistema Nacional de Salud. [accedido 15 junio 2020]. Disponible en: https://www.boe.es/boe/dias/2019/01/25/pdfs/BOE-A-2019-856.pdf.

63. von Mackensen S, Myrin Westesson L, Kavakli K, Klukowska A, Escuriola C, Uitslager N, et al. The impact of psychosocial determinants on caregivers’ burden of children with haemophilia (results of the BBC study). Haemoph Off J World Fed Hemoph. 2019;25(3):424-32, doi: 10.1111/hae.13684.

64. Silva Romero I, López-Chicheri B, Myrén K-J, Meritxell A, Darbà J. Análisis del impacto económico de Elocta® (rFVIIIFc) en el tratamiento a demanda y en profilaxis de la hemofilia a en España. XXXVII Jornadas de Economía de la Salud; 2017.

65. Instituto Nacional de Estadística. Encuesta de presupuestos familiares. Base 2006. Gasto por códigos de gasto (5 dígitos). Gasto medio por persona y año en juegos y hobbies. INE. [accedido 16 junio 2020]. Disponible en: https://www.ine.es/jaxiT3/Tabla.htm?t=24882&L=0.

66. López Bastida J, Linertová R, Serrano Aguilar P, Hens Pérez M, Posada de la Paz M, Oliva Moreno J. Los costes socioeconómicos y la calidad de vida relacionada con la salud en pacientes con enfermedades raras en España. 2012.

67. Dutreil S, Rice J, Merritt D, Kuebler EJ. Parents Empowering Parents (PEP) Program: understanding its impact on the bleeding disorders community. Haemophilia. 2011;17(5):e895-900, doi: 10.1111/j.1365-2516.2011.02512.x.

68. Ministerio de Cultura y Deporte. Anuario de Estadísticas Deportivas 2020. 2020.

69. Organización de Consumidores y Usuarios. ¿Cuánto cuesta empezar el curso? www.ocu.org. [accedido 22 junio 2020]. Disponible en: https://www.ocu.org/consumo-familia/bebes/informe/gastos-escolares-2017-18#.

70. Gringeri A, Mackensen SV, Auerswald G, Bullinger M, Garrido RP, Kellermann E, et al. Health status and health-related quality of life of children with haemophilia from six West European countries. Haemophilia. 2004;10(s1):26-33, doi: 10.1111/j.1355-0691.2004.00876.x.

71. Thies KM, McAllister JW. The Health and Education Leadership Project: A School Initiative for Children and Adolescents with Chronic Health Conditions. J Sch Health. 2001;71(5):167-72, doi: 10.1111/j.1746-1561.2001.tb07309.x.

72. Krishnan S, Vietri J, Furlan R, Duncan N. Adherence to prophylaxis is associated with better outcomes in moderate and severe haemophilia: results of a patient survey. Haemophilia. 2015;21(1):64-70, doi: 10.1111/hae.12533.

73. Limperg PF, Joosten MMH, Fijnvandraat K, Peters M, Grootenhuis MA, Haverman L. Male gender, school attendance and sports participation are positively associated with health-related quality of life in children and adolescents with congenital bleeding disorders. Haemoph Off J World Fed Hemoph. 2018;24(3):395-404, doi: 10.1111/hae.13420.

74. Geraghty S, Dunkley T, Harrington C, Lindvall K, MaHAs J, Sek J. Practice patterns in haemophilia A therapy – global progress towards optimal care. Haemophilia. 2006;12(1):75-81, doi: 10.1111/j.1365-2516.2006.01189.x.

1. The three bars (///) in each final proposal indicate the integration of related proposals made by the different working groups of the Multidisciplinary Working Group. [↑](#footnote-ref-1)
2. The three bars (///) in each final proposal indicate the integration of related proposals made by the different working groups of the Multidisciplinary Working Group. [↑](#footnote-ref-2)
3. The three bars (///) in each final proposal indicate the integration of related proposals made by the different working groups of the Multidisciplinary Working Group. [↑](#footnote-ref-3)
4. The three bars (///) in each final proposal indicate the integration of related proposals made by the different working groups of the Multidisciplinary Working Group. [↑](#footnote-ref-4)
